# Metal Toxicity at the Synapse: Presynaptic, Postsynaptic, and Long-Term Effects

**DOI:** 10.1155/2012/132671

**Published:** 2012-01-12

**Authors:** Sanah Sadiq, Zena Ghazala, Arnab Chowdhury, Dietrich Büsselberg

**Affiliations:** Weill Cornell Medical College in Qatar, Qatar Foundation—Education City, P.O. Box 24144, Doha, Qatar

## Abstract

Metal neurotoxicity is a global health concern. This paper summarizes the evidence for metal interactions with synaptic transmission and synaptic plasticity. *Presynaptically* metal ions modulate neurotransmitter release through their interaction with synaptic vesicles, ion channels, and the metabolism of neurotransmitters (NT). Many metals (e.g., *Pb* ^2+^, *Cd* ^2+^, and *Hg* ^+^) also interact with intracellular signaling pathways. *Postsynaptically*, processes associated with the binding of NT to their receptors, activation of channels, and degradation of NT are altered by metals. *Zn* ^2+^, *Pb* ^2+^, *Cu* ^2+^, *Cd* ^2+^, *Ni* ^2+^, *Co* ^2+^, *Li* ^3+^, *Hg* ^+^, and methylmercury modulate NMDA, AMPA/kainate, and/or GABA receptors activity. *Al* ^3+^, *Pb* ^2+^, *Cd* ^2+^, and *As*_2_ *O*_3_ also impair *synaptic plasticity* by targeting molecules such as CaM, PKC, and NOS as well as the transcription machinery involved in the maintenance of synaptic plasticity. The multiple effects of metals might occur simultaneously and are based on the specific metal species, metal concentrations, and the types of neurons involved.

## 1. Introduction

Metals and their compounds are distributed in ecosystems as a result of natural processes as well as anthropogenic activities. Metals are used in their elementary form as well as in compounds for various human needs. Therefore, a number of these metals enter our environment as a consequence of their widespread use in preservatives, biocides, and paints [1]. They are taken up by organisms through inhalation or by ingestion of food and water contaminated with these metals. For living systems, metals can be divided in those which are essential for life, such as cobalt (Co), copper (Cu), zinc (Zn), manganese (Mn), and iron (Fe); which are potentially toxic only at higher concentration, and those which have no known biological function, which can be toxic at all concentrations such as cadmium (Cd), chromium (Cr), mercury (Hg), and* lead *(Pb) [13] (for all abbreviations used in the review please refer to the abbreviations section; to facilitate reading, the names of the specific metals discussed are given in *italics*). 

Since the uptake mechanisms of the body are not able to distinguish between “physiologically required” and harmful metals, the toxic metals absorbed consequently might interact with the functions of the central nervous system (CNS), liver, kidneys, and hematopoietic system, thus presenting a significant health hazard. In this review, we will examine the effects of these metals in the CNS, specifically at the synapse.

The human brain has about 10^11^ neurons, which interconnect and “communicate” with each other through synapses. It is estimated that each neuron has approximately 7000 synapses. At the presynaptic side of the synapse the incoming electrical signal, in from of action potentials, is transformed to a chemical signal in the form of neurotransmitter release. Synaptic transmission depends on the timely opening of membrane channels, the precise functioning of intracellular signaling pathways, and metabolic pathways involved in the synthesis and the release of neurotransmitters. Postsynaptically the binding of neurotransmitters changes the membrane potential, resulting in a hyper- or depolarization of the neuron and in the generation of an action potential when the threshold potential is reached. These are crucial process and the basis of all higher cognitive functions including learning and memory.

Therefore, we highlight the mechanisms by which metals and their compounds interfere with the processes of synaptic transmission and synaptic plasticity. This review covers the effects of metals on signal transmission from the presynaptic to the postsynaptic membrane, as well as the effects on synaptic plasticity with an emphasis on learning and memory, since subtle alterations in synaptic transmission due to the interaction of metals may have profound toxic effects in the CNS [14]. 

Some metals, which have already been shown to alter synaptic transmission, are discussed in this review. The metals are listed in an alphabetical order below with a short description of their neurotoxic effects, to show their relevance to this study (for more details regarding the neurotoxicity of these metals see [15]). 

*Aluminum* was found present in high concentrations in brains of patients with Alzheimer's disease, Parkinson's disease, and dialysis encephalopathy and could contribute to neurogenerative disorders [21]. In animals the administration of *aluminum* salts results in neurofibrillary degeneration, a condition similar to the encephalopathy in Alzheimer's disease [3]. 

*Arsenic*, one of the oldest known poisons, due to its cholera-like symptoms, became a favorite poisoning agent and earned the title the “Poison of Kings” [15]. An acute ingestion of *arsenic* affects many systems of the body including gastrointestinal, cardiovascular, respiratory, and the nervous system. Even today, chronic low-dose exposure to *arsenic* is very common in countries like Bangladesh, India, Taiwan, and other parts of South East Asia due to contamination of groundwater by *arsenic*. It is a major cause of infant mortality in Bangladesh [8]. Chronic manifestations of *arsenic* poisoning are pigmentation changes, gastrointestinal problems, anemia, liver disease, black foot disease, and Mees' lines on the nails. Central neuropathy due to *Arsenic* poisoning usually manifests as impairment of learning, short-term memory and concentration. However, peripheral neuropathy is more frequently observed and this might last for several years. It manifests as a rapid and severe ascending weakness and sometimes these patients require mechanical ventilation [8, 15].

*Cadmium* and *manganese* also have neurotoxic effects, where *cadmium* damages cells of the cerebellar cortices of young rats as well as rabbits and chronic *manganese* poisoning causes extrapyramidal symptoms much like those of Wilson's disease and Parkinsonism [9]. Moreover, increased total *cadmium* levels in human hair were associated with mental retardation and impairment in visual motor abilities [4]. Similar toxicities also occur in humans.

*Lead*, whose mechanisms of neurotoxicity have been extensively studied, was discovered more than 5000 years ago and was used in the ancient world for lead water piping, as utensils, to sweeten food and wine, and as a constituent of eye paints [2]. It was discovered that acute exposure to *Pb* could cause lead colic and mental disturbances and even chronic exposure to low concentrations of lead in children caused several cognitive and behavioral disturbances. Since *Pb* crosses the placenta, prenatal exposure to lead can have especially severe consequences [4, 5, 15]. 

Exposure to dietary *methylmercury* leads to Minamata disease, which manifested in patients as paresthesias followed by irreversible impairment of vision, hearing, speech, gait, and ultimately leads to death. In addition, cognitive impairment ensued with prenatal exposure to *methylmercury *[4]. 

*Organo-tins* are industrially produced in large quantities for applications as PVC stabilizers, glass coverings, silicone, wood preserver additives, and antifouling paints. Moreover, considerable amounts of *organo-tins* are released in the environment causing large concern about their impact on human health. Due to their lipophilicity *organo-tins* are taken up by humans and distributed in different tissues. In mammalian organs such as brain, liver, and kidneys, *organo-tins* are biotransformed and this process may increase their toxicity [7]. Specifically *alkyl-tins* have been shown to cause neurotoxicity [15]. 

Even though metals are well known for their various toxicities, they are also used as therapeutic agents. *Lithium* salts have been used in the treatment and prophylaxis of bipolar affective disorder [10, 16]. *Arsenic* in the form of *arsenic trioxide* is used for the treatment of leishmaniasis, leukemia, and trypanosomiasis [8, 15]. The specific toxicities of some metals are actually being used to man's benefit, especially for the treatment of cancers.* Cisplatin* (*cis-diammine-dichloro-platin = CDDP*) is used as an anticancer drug and testicular cancer, endometrial cancer, prostatic tumors, bladder carcinoma, and small cell bronchial carcinoma [17] are successfully treated with this drug.

With the wide description of harmful effects of metals as well as their irreplaceability in modern life and medicine, it becomes essential to demarcate the level at which metals become toxic. This includes concentrations of metals as well as their targets of actions. Recognizing the targets sites at which metals interact can serve as a stepping-stone for the development of therapeutic agents to counteract metal toxicity as well as the side effects of anticancer drugs such as *arsenic* and *cisplatin* compounds. 

This paper aims to review the literature available of the mechanisms of actions of metals at targets presynaptically, postsynaptically, and on long-term potentiation (LTP) and summarizes the findings in a logical and easily comprehensible manner. In the first part the toxic effects of organic and inorganic metals on the ***pre**sy*naptic part will be described (Section 2), followed by a review of their ***post**s*ynaptic actions (Section 3), and the review finally looks at the impairment of ***synaptic plasticity*** (Section 4) before concluding remarks are made (Section 5).

## 2. Presynaptic Targets of Toxic Metals

**Figure 1:**
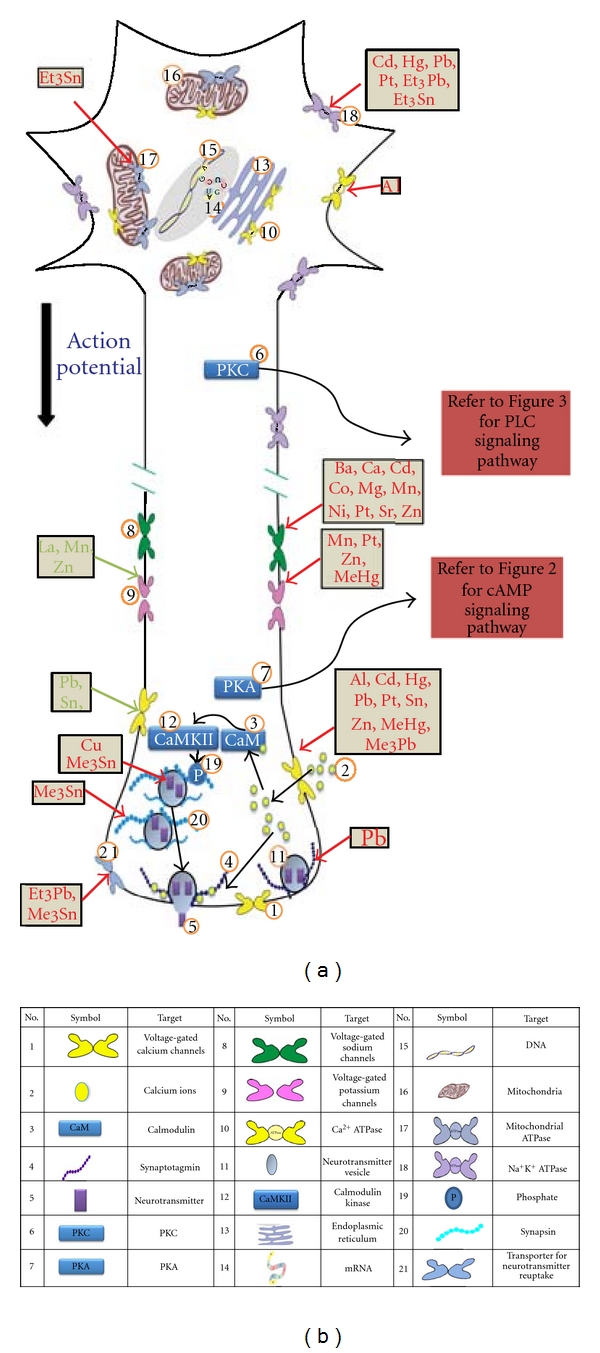
Presynaptic targets of neurotoxic metals. Events at the synapse from the arrival of the action potential which results in the membrane depolarization-induced opening of voltage-activated calcium channels and the entry of calcium which activates CaM, which activates CaM kinases and causes the phosphorylation of synaptic vesicle-associated proteins and an increase in readily releasable neurotransmitter vesicles. Calcium also binds synaptotagmin and causes exocytosis of neurotransmitter from the vesicles. Shown in boxes are the metals and the targets at which they act in the synaptic transduction pathway. A table at the end indicates the symbols and what they indicate. Green indicates activation or upregulation while red indicates inhibition or downregulation. Please refer to the section of Abbreviations and metals.

Presynaptically, the action potential, which is an electrical signal, is transduced to a chemical signal in the form of neurotransmitter release. Generally, the action potential induces a membrane depolarization, which opens voltage gated calcium channels allowing the influx of Ca^2+^. Ca^2+^ activates calmodulin (CaM) and therefore CaM kinases (CamK) are activated, which leads to the phosphorylation of synaptic vesicle associated proteins and the conversion of the reserve pool of synaptic vesicles to a readily releasable pool of vesicles. Ca^2+^ also binds synaptotagmin, a calcium sensor protein in the vesicle membrane and triggers neurotransmitter vesicle fusion and the release of neurotransmitter [18] (Figure 1).

Metals interact with specific targets in these pathways and the same metals might even interact with various targets simultaneously. For instance, *aluminum* blocks voltage gated calcium channels, decreases the biological activity of CaM, and also inhibits Ca^2+^ ATPase [19, 20]. In addition, if a metal interacts with an upstream target of a pathway, it may influence all the processes succeeding it. For example, *cadmium* reduces voltage activated calcium channel currents, therefore, it can influence the intracellular calcium concentration and consequently the activation of CaM and calcium-dependent intracellular signaling pathways [6, 11]. Notably, *cadmium* caused a decrease in release of excitatory neurotransmitters glutamate and aspartate while it caused an increase in the release of inhibitory neurotransmitters GABA and glycine [12].

The upcoming sections (Sections 2.1 to 2.6) highlight the literature relating to the toxic effects of metals on presynaptic targets including voltage-activated ion channels (Section 2.1), signaling cascades (Section 2.2), transporters (Section 2.3), synaptic vesicle associated proteins (Section 2.4), neurotransmitters (Section 2.5), and neurofilaments and microtubules (Section 2.6). For ease of access, wherever possible metals are described in alphabetical order in each Section.

### 2.1. Voltage-Activated Channels

#### 2.1.1. Voltage-Activated Calcium Channels

Voltage activated calcium channels open by a depolarization. They are subdivided into high- and low-voltage activated channels. The high-voltage activated channels, which have to be depolarized to more positive voltages than −30 mV for activation, include the L-type, P/Q-type, N-type, and R-type, where the L-type has a “long-lasting” current. The other types are divided on the basis of their inactivation and their susceptibility to various peptide toxins. There are also low-voltage activated channels which are mainly composed of the T-type channels which have a small, fast inactivating and therefore transient current [22].

*Aluminum (Al^3+^)* blocked N- and L-type voltage activated calcium channels in cultured rat dorsal root ganglions, with a threshold concentration of 20 *μ*M and a Hill's coefficient of 3 (Table 1). It also required an open channel for its actions thereby indicating that the possible site of action of *Al^3+^* was inside the channel. The current-voltage relation was shifted to depolarizing voltages in the presence of *Al^3+^* [19]. *Aluminum* also blocked voltage-activated calcium channels *in vivo* in rats when given 10 mg per kg body weight per day intraperitoneally for 4 weeks. Inhibition was nearly 85% in the corpus striatum, 58% in the cerebral cortex, and 46% in the hippocampus [20].

*Cadmium (Cd^2+^)* effectively reduced voltage-activated calcium channel currents, which were high threshold and fast inactivating types in cultured chick dorsal root ganglion cells, at concentrations of 20 *μ*M. This block was released at hyperpolarizing voltages, which may be due to shifts in gating and permeability of the channels. When the membrane potential was hyperpolarized, the channels conducted transiently, as *Cd^2+^* exited the channels, but closed again thereafter. The channels can close with *Cd^2+^* in the channel pore, therefore implying that *Cd^2+^* does not affect the closing mechanisms of the channels [6]. Similar results were obtained in squid giant fiber neurons. In addition, a kinetic model was created and the binding site for *Cd^2+^* was determined to be near the outer end of the pore, and the entry of *Cd^2+^* into the pore was voltage independent while its exit was voltage dependent [11].

*Lead (Pb^2+^)* is a potent blocker of voltage-activated calcium channels in invertebrate *Aplysia* neurons as well as in mammalian neurons [19, 23, 25, 27, 28, 30]. There is no change in the voltage dependence of activation or inactivation of the channels in mammalian neurons, which suggests an external binding site for *Pb^2+^* [25, 27, 28]. In mammals *Pb^2+^* blocked N-, L- and T-type voltage activated calcium channels [19, 27, 28, 30]. The block of L- and T-type channels was concentration dependent and reversible in N1E-115 mouse neuroblastoma cells [30]. The concentration for 50% inhibition (IC_50_) of L-type channels was 30 nM, and for N-type channels it was 80 nM free *Pb^2+^* where 10 mM Ba^2+^ was used as the charge carrier in cultured E-18 rat hippocampal neurons [28]. Some contradictory data were obtained in a study of mouse N1E-115 neuroblastoma cells, where in five of the fifteen cells studied, 2.3 *μ*M *Pb^2+^* enhanced L-type calcium channel currents and also enhanced the inactivation of L-type channels at holding potentials of −60 to −40 mV [30]. A study on human neuroblastoma cells SH-SY5Y determined that *lead acetate* at concentrations of 1 to 30 *μ*M blocked voltage-activated calcium channels, both N- and L-types in a concentration-dependent and reversible way. More importantly, the concentrations used in the study were inclusive of the blood level concentrations at which children present with neuropsychological disorders (between 1.5–2.5 *μ*M) [29]. 

*Mercury* (*Hg^2+^*) blocked voltage-activated calcium channels with an IC_50_ of 1.1 *μ*M *in vitro* in rat pup dorsal root ganglion cells, and it required a partially open channel for its block [24]. *Mercury *(*Hg^2+^*) blocked neuronal N- and R-type calcium channels transiently expressed in human embryonic kidney 293 cells with an IC_50_ of 2.2 and 0.7 *μ*M. This effect was partially reversible in N-type but not in R-type channels [26]. *Mercury* also blocked T-type calcium channel currents in the concentration range of 0.5–2 *μ*M in cultured rat dorsal root ganglion cells. In addition the current-voltage relation was shifted to positive voltages implying that *mercury* may have an effect on channel gating [40].

*Platinum *in the form of *cis-diammine-dichloro-platin (CDDP)* reduced voltage-activated calcium channel currents in dorsal root ganglion cells of rats *in vitro*. *CDDP* reduced peak *calcium* current with an IC_50_ of 23.9 ± 4.5 *μ*M and sustained current with an IC_50_ of 38.8 ± 6.1 *μ*M in small neurons with a diameter of ≤20 *μ*m. Surprisingly, in large neurons with a cross-sectional diameter of ≥25 *μ*m, the peak calcium current was only reduced by 14.1 ± 2.3% even with a concentration of 100 *μ*M *CDDP*. It is unlikely that the voltage-activated calcium channel currents were blocked directly since the small and large cells were unequally affected and the Hill's coefficient was not 1.* CDDP* probably decreases voltage-activated calcium channel currents by acting through an intracellular pathway more prominent in small neurons, possibly through IP_3_ receptor activation as described later [17].

*Tin (Sn^2+^)* used as *stannous chloride (SnCl_2_) *decreased voltage-activated calcium channel currents *in vitro *in rat dorsal root ganglion cells in a concentration-dependent manner with a threshold of 1 *μ*M. These effects were found to be irreversible [41]. However, contradictory results were obtained in a study of motor nerve terminals of frog, where nerve muscle preparations were exposed to 50 *μ*M *SnCl_2_*, which caused an increased inward Ca^2+^ current [34].

*Zinc (Zn^2+^)* blocked voltage-activated calcium channels in cultured rat dorsal root ganglion cells [19, 24, 31]. The IC_50_ for this effect on N- and L-type channels was 69 *μ*M *Zn^2+^* while the Hill's coefficient was 1. T-type currents were more sensitive, and the block was partly reversible in 50% of the neurons [31]. *Zinc* did not require an open channel for this blocking effect [24]. The current voltage relationship shifted to more depolarizing voltages in the presence of *Zn^2+^*, implying that the mechanism of action of *Zn^2+^* may involve the screening of charges in the vicinity of the channels [19].

*Methylmercury (MeHg)* caused an increase in calcium influx and therefore [Ca^2+^]_i_ through nifedipine and *ω*-conotoxin sensitive mechanisms, that is, through either, L-, N-, or Q-type calcium channels [36]. However, *methylmercury* caused an irreversible time and concentration dependent block of calcium channel currents at concentrations between 0.25 and 1 *μ*M *in vitro* in rat cerebellar granule neurons. The block did not require depolarization, indicating that it did not require an open channel. Increasing the frequency of stimulation of cells increased the magnitude of block at 0.25 *μ*M and 0.5 *μ*M but not at 1 *μ*M, which may suggest the presence of other counteracting effects. None of the calcium channel antagonists used—*ω*-conotoxin GVIA, *ω*-conotoxin MVIIC, *ω*-agatoxin IVA, calcicludine, and nimodipine, were able to decrease the *MeHg*-induced block of calcium channel currents [38]. *MeHg* blocked N-, R-, and L-type voltage-activated calcium channels [26, 39]. *MeHg* blocked human neuronal N- and R- type calcium channel currents transiently expressed in human embryonic kidney 293 cells with an IC_50_ of 1.3 *μ*M and 1.1 *μ*M respectively (Table 4). This block was determined to be irreversible [26]. 

*Trimethyl lead* blocked voltage-activated calcium channels with a threshold concentration of 0.5 *μ*M *in vitro *in rat dorsal root ganglion cells. This block was irreversible and concentration dependent but not voltage dependent. It required an open channel and the IC_50_ was between 1–5 *μ*M [33].

#### 2.1.2. Voltage-Activated Sodium Channels

There are 9 subtypes of voltage-activated sodium channels Na_v_ 1.1–Na_v_ 1.9 distinguished by their sensitivity to tetrodotoxin and their rate of inactivation. Na*_v_* 1.8 and Na*_v_* 1.9 have relatively slower inactivation [35]. Na*_v_* 1.1, Na*_v_* 1.2, Na*_v_* 1.3, and Na*_v_* 1.7 are expressed in neurons and are highly sensitive to tetrodotoxin. Na*_v_* 1.5, Na*_v_* 1.8, and Na*_v_* 1.9 are relatively tetrodotoxin resistant and are found in heart and dorsal root ganglion neurons. Na*_v_* 1.4 and Na*_v_* 1.6 are mostly expressed in skeletal muscle and the CNS, respectively [37].

*Zinc (Zn^2+^)* and *cadmium (Cd^2+^)* reduced both tetrodotoxin-sensitive and tetrodotoxin-insensitive voltage-activated sodium channel currents in voltage clamp experiments in neuronal, cardiac, and skeletal muscle cells [42]. Tetrodotoxin-resistant channels were more sensitive to *Zn^2+^* and *Cd^2+^* with IC_50_ of the block being 50 *μ*M and 0.2 mM, respectively; tetrodotoxin-sensitive channels were less resistant with IC_50_ of the block being 2 mM and 5 mM for *Zn^2+^* and *Cd^2+^*, respectively [42] (compare effects in Table 1). It was suggested that the site of action of *Zn^2+^* contains cysteine sulfhydryl groups in or near the saxitoxin binding site since Zn^2+^ was able to relieve the saxitoxin-induced block of the channel in a competitive manner, and the blocking action of *zinc* was inhibited by sulfhydryl-specific alkylating reagents. These experiments were done in voltage-activated sodium channels taken from the hearts of dogs or calves [32]. 

*SnCl_2_* had an effect on voltage-activated sodium channel currents of the mollusk *Lymnaea stagnalis in vitro* where it shifted the current voltage curve to the left. SnCl_2_ increased voltage-activated sodium channel currents at a concentration of 10 *μ*M, but caused a depression in current at concentrations above 25 *μ*M. Organic tin in the form of *(CH_3_)_3_SnCl* decreased significantly the Na current only at high concentrations above 100 *μ*M. Additionally the current voltage curve was shifted to the left. These effects were time dependent and irreversible [43]. 

*Cobalt*, *manganese*, *nickel*, *calcium*, *magnesium*, *strontium,* and *barium* in divalent cation form blocked both tetrodotoxin-sensitive and tetrodotoxin-insensitive channels in channels incorporated into planar bilayers in the presence of batrachotoxin. The block was voltage dependent and the sequence of affinity to block was *Co^2+^*≅* Ni^2+^*>*Mn^2+^*>* Ca^2+^*>* Mg^2+^*>* Sr^2+^*>* Ba^2+^. *The suggested mechanisms of block included a specific divalent cation binding site and surface charge screening [44]. Also 10 *μ*M of the anticancer drug *CDDP* reduced voltage-activated sodium channel currents by 9.2% ± 7.2% in rat dorsal root ganglions *in vitro *[17].

#### 2.1.3. Voltage-Activated Potassium Channels

The family of voltage-activated potassium channel includes K_v _1–6, K_v_ 8, and K_v_ 9, where the principal subunit of the channels contains 6 transmembrane domains. All these channels are expressed in brain tissue [45]. Whole cell patch-clamp measurements of transient voltage-dependent potassium currents in rat suprachiasmatic nucleus neurons showed that *Zn^2+^* potentiated current when activated from a holding potential of −60 mV (approximately the resting membrane potential). This potentiation was voltage dependent and arose from a shift of the inactivation current to more positive voltages. *Zn^2+^* (30 *μ*M) shifted the half-inactivation voltage by 20 mV from −80 mV to −60 mV [46]. Kuo and Chen showed that at hyperpolarized voltages *Zn^2+^* inhibited voltage-dependent transient K^+^ currents which can be accounted for by the selective binding of *Zn^2+^* to closed K^+^-channels with a dissociation constant of approximately 3 *μ*M, which kept the channels closed and slowed the activation of the current [47].

Whole cell patch clamp studies in central neurons of Drosophila third instar larvae showed that millimolar *Ca^2+^* and Mg^2*+ *^ concentrations and micromolar concentrations of *Zn^2+^* increased the peak inactivation current and shifted the steady-state inactivation curve of voltage gated potassium channels to more positive voltages, but had no effect on the voltage dependence of activation. A micromolar concentration *Cd^2+^* had the same effect; however, millimolar concentrations of *Cd^2+^* had an effect on both steady state inactivation and activation curves, where the midpoint of the activation curve was shifted more positively. The potency of effect on the inactivation current in terms of amount of shift of steady state inactivation curves was *Zn^2+^* (2 mM) >*Cd^2+^* (2 mM) >*Ca^2+^* (20 mM) >*Mg^2+^* (20 mM). The mechanism of action was most likely through specific binding to the channels at extracellular sites [48].

10 *μ*M* cisplatin* in the form of *CDDP* reduced voltage-gated potassium channel currents by 20.9 ± 4.8% in small dorsal root ganglion neurons while 100 *μ*M *CDDP* reduced the peak current by 12.8 ± 3.4% [17]. Micromolar concentrations of *lanthanum (La^3+^)* enhanced outward voltage-gated potassium channel currents evoked by depolarization steps from −50 mV in rat cerebellar granule neurons. 10 *μ*M *La^3+^* shifted the steady state inactivation curve by approximately 40 mV in the depolarizing direction and increased the slope factor slightly [49].

Mayer and Sugiyama showed that fast activating transient potassium channel currents were reduced by 10 mM *manganese* (*Mn^2+^*) in cultured rat sensory neurons. This reduction was due to a depolarizing shift of the activation curve and a slight reduction in maximum conductance. At the same concentration, steady state inactivation curves were also shifted to depolarizing voltages. The positive shift of steady state inactivation and activation curves were obtained for other metals as well, where the potency of shift was *Cd^2+^*  >*Mn^2+^ = Co^2+^*  >*Ca^2+^*  >* Mg^2+^. *Lower concentrations of *Mn^2+^* (1 mM), however, increased the amplitude of fast inactivating transient potassium channel currents at prepulse potentials from −50 to −70 mV, which was due to a shift of the inactivation curve with no significant shift in the activation curve. These effects may have been due to binding to a specific site within the channel or to phospholipids in close proximity of the gating apparatus [50].

Organic metals also affect voltage-gated potassium channels. *Methylmercury (MeHg)* blocked voltage-gated potassium channels irreversibly, with an IC_50_ of 2.2 ± 0.3 *μ*M in a concentration-dependent manner. The Hill's coefficient for this block was *∼*1 [51].

### 2.2. Signaling Cascades

#### 2.2.1. The cAMP System

**Figure 2:**
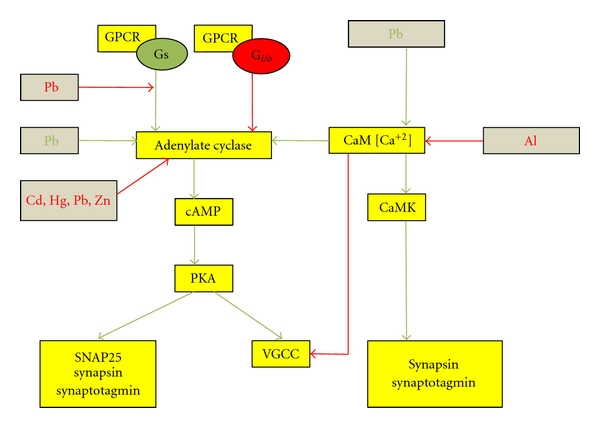
Effects of metals on the PLC signaling pathway at presynaptic terminal (green: activation/increase, red: inhibition/decrease). The PLC system consists of GPCRs coupled to Gq, which activate PLC, which activates DAG and IP_3_. IP_3_ increases intracellular calcium ([Ca^2+^]_i_) and activates DOC2 and synaptotagmin which leads to increased evoked release and readily releasable pool size. DAG activates PKC, which activates voltage-gated calcium channels. PKC phosphorylates Munc-18, which negatively regulates vesicle fusion and syntaxin. PKC activation leads to the increased spontaneous and evoked neurotransmitter release. The effects of metals on this pathway are shown in this figure where a green color indicates an activation/upregulation and a red color indicates an inhibition/downregulation.

G-protein-coupled receptors (GPCR) are coupled to G_s_ or G_i/o_, where G_s_ acts as a stimulator of adenylate cyclase and the G*α* subunit of G_i/o_ acts as an inhibitor of PKA (Figure 1). PKA phosphorylates Ca^2+^-channels, thereby enhancing the influx of Ca^2+^ and this increases the release of neurotransmitters [52]. Also PKA phosphorylates SNAP-25 and this leads to a larger pool of readily releasable vesicles [52]. The cAMP-system appears to enhance the release of neurotransmitter in response to a stimulus (Figure 2).

*In vitro* and *in vivo* exposure to *lead acetate* decreased cAMP-dependent synaptic vesicle protein phosphorylation in rat brain which is most likely a contributing mechanism of *lead* toxicity [53].

**Gs**

Rodrigues and colleagues determined the effect of *lead acetate *on rat cerebral cortex membranes using 5′ Guanylylimidodiphosphate (Gpp(NH)p). Gpp(NH)p is a nucleotide phosphorylase-resistant GTP analogue, which is known to stimulate adenylate cyclase by saturating Gs. On preincubation of membranes with *lead acetate*, the stimulatory effect of Gpp(NH)p on the adenylate cyclase activity was inhibited [10].

**Adenylate Cyclase**

The same group [10] also determined the effects of *lead* acetate on adenylate cyclase activity in the cerebral cortex membranes and found that *lead* caused a concentration-dependent inhibition of adenylate cyclase activity with an IC_50_ of 2.5 ± 0.1 *μ*M (Table 2) [10].

In another series of experiments, Ewers and Erbe [54] determined the effects of *lead, cadmium, and mercury* on adenylate cyclase of the cerebrum, cerebellum, and the brain stem, *in vitro* and *in vivo*. Adenylate cyclase activity was determined in terms of the number of moles of cAMP formed. Concentrations between 0.1 and 30 *μ*M *lead nitrate*, *cadmium nitrate, *or *mercury nitrate* inhibited adenylate cyclase activity *in vitro* in homogenates of the cerebrum, brain stem, and the cerebellum. *In vivo* studies were performed on rats, which received *lead acetate* dissolved in sterile demineralized water, cAMP formation was determined 1 hour, 4 hours, and 24 hours after treatment. In the cerebellum, and brainstem, adenylate cyclase activity increased after one hour by about 25% but was unaffected in the cerebrum. After four hours, adenylate cyclase declined by 29%, 33%, and 21% in the cerebrum, cerebellum, and brainstem respectfully. By 24 hours adenylate cyclase activity had returned to normal in the cerebrum and brainstem but not in the cerebellum [54]. These differences in the effects of *lead* acetate on adenylate cyclase in different parts of the brain may be an indicator of the varied effects of *lead* on different isoforms of adenylate cyclase.

*Zinc* (*Zn^2+^*) inhibited adenylate cyclase with an IC_50_ of 1-2 *μ*M and a Hill's coefficient of 1.33, which was not competitive with *Mg^2+^* or *Mg^2+^*ATPase [82]. Both the CI and the CII domains of adenylate cyclase bind *Zn^2+^* with high affinity which is correlated with *Zn^2+^* inhibition of enzyme activity [83].

#### 2.2.2. The PLC System

**Figure 3:**
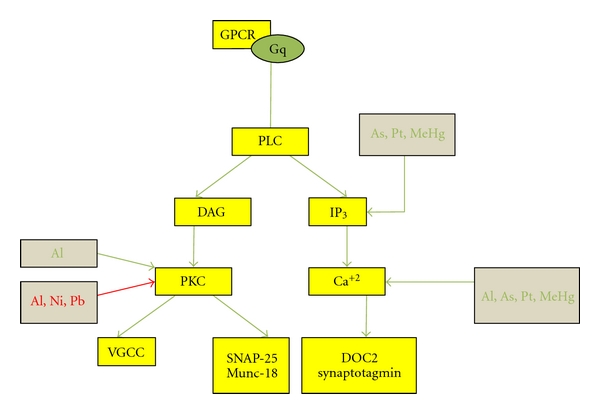
Effects of metals on the PLC signaling pathway at presynaptic terminal (green: activation/increase, red: inhibition/decrease). The PLC system consists of GPCRs coupled to Gq, which activate PLC, which activates DAG and IP_3_. IP_3_ increases intracellular calcium ([Ca^2+^]_i_) and activates DOC2 and synaptotagmin which leads to increased evoked release and readily releasable pool size. DAG activates PKC, which activates voltage-gated calcium channels. PKC phosphorylates Munc 18, which negatively regulates vesicle fusion and syntaxin. PKC activation leads to the increased spontaneous and evoked neurotransmitter release. The effects of metals on this pathway are shown in this figure where a green color indicates an activation/upregulation and a red color indicates an inhibition/downregulation.

The PLC system consists of GPCRs coupled to Gq, which activate DAG and IP_3_ through PLC. IP_3_ causes an increase of intracellular calcium ([Ca^2+^]_i_) and the activation of DOC2 and synaptotagmin which leads to increased evoked release and readily releasable pool size. DAG through PKC causes an activation of voltage-gated calcium channels. PKC phosphorylates Munc 18, which negatively regulates syntaxin and synaptic vesicle fusion [52, 84]. PKC activation eventually leads to an increase of spontaneous and evoked neurotransmitter release and more readily releasable pool of vesicles (Figure 3) [52].

**PKC**

Metals that inhibit PKC include *lead, aluminum, *and* nickel. Pb^2+^* inhibits PKC enzymes through interactions with its catalytic domains [85]. The effect of *aluminum* on PKC is debated: Julka and Gill demonstrated that *aluminum* lactate given to male albino rats for four weeks, caused an inhibition of PKC at all concentrations used (up to 100 *μ*M). This was shown both *in vivo *and *in vitro. *The largest inhibition was observed in the cerebral cortex (47.73%) followed by the hippocampus (45.95%) and the corpus striatum (38.74%) [20]. However, contrasting findings were determined by Johnson and coworkers who showed that *aluminum* sulfate, when given orally for a period of 4 months to male Sprague-Dawley rats, showed an increase in PKC specific activity by 60% and total activity by 70% in the soluble fraction of cerebral cortex homogenates [69]. The different effects of *aluminum* could be attributed to the mode of intake reflecting differences in concentration of *aluminum* absorbed and its distribution to the brain or the duration of exposure.

Microarray analysis in neuronal PC12 cells indicated that exposure to *Nickel* (Ni^2+^) caused a decline in the transcription of two isoforms of PKC- prkcc, prkz, and two regulatory binding proteins prkcbp1 and prkcdbp, and also caused temporary upregulation and downregulation of prkcq at 24 hours and 72 hours, respectively [86]. These effects are important in terms of the events at the synapse because PKC activates voltage-activated calcium channels, and increases the secretion of neurotransmitter through effects on proteins involved in neurotransmitter exocytosis- Munc-18, and SNAP25 (a SNARE protein) [87].

**IP_3_**

Increase of [Ca^2+^]_i_ in human cervix adenocarcinoma cells by *cisplatin* (0.001–10 *μ*M) was dependent on extracellular Ca^2+^ and was blocked by an IP_3_ receptor blocker. The types 1–3 IP_3_ receptors were at the cellular membrane of these cells, which suggests a possible mechanism of *cisplatin*-induced calcium entry through IP_3_ receptor activation. This was supported by the observation that the same results were not obtained in human osteosarcoma cells, which in addition did not show the presence of types 1–3 IP_3_ receptors at cell membrane [88]. *Arsenic trioxide (As_2_O_3_)* similarly caused an increase in intracellular calcium which was dependent on calcium release from the intracellular calcium stores through the activation of IP_3 _receptors [88]. *MeHg* also causes an increase in intracellular calcium, which may be due to release from intracellular stores through inositol phosphate. *MeHg* doubled intracellular inositol phosphate levels at concentrations above 3 *μ*M *in vitro* in rat cerebellar granule neurons [89].

#### 2.2.3. Intracellular Calcium ([Ca^2+^]_i_)

*Aluminum (Al^3+^)* caused an increase of [Ca^2+^]_i_ in rat synaptosomes, which could be a consequence of the inhibition of the Ca^2+^-ATPase [20]. 1 *μ*M *arsenic* trioxide (As_2_O_3_) caused an irreversible increase in [Ca^2+^]_i_ in human neuroblastoma cells (SY-5Y) and in human embryonic kidney 293 cells. This rise of [Ca^2+^]_i_ was independent of extracellular calcium, but dependent on intracellular calcium stores. Blocking of IP_3_ receptor and ryanodine receptors with their specific blockers reduced the increase in [Ca^2+^]_i_ indicating their involvement in this process [88]. *Cisplatin* also increased [Ca^2+^]_i_ in a concentration-dependent manner in human cervix adenocarcinoma cells but not in human osteosarcoma cells. It is unlikely that the increase in [Ca^2+^]_i_ is induced by entry of extracellular calcium, but more likely through activation of IP_3_ receptor as described above [88]. 

In addition, [Ca^2+^]_i_ could indirectly be affected by several mechanisms. For example, *[cis-(NH_3_)_2_Pt(H_2_O)_2_]^2+^*, a form of platinum, caused an uncoupling of oxidative phosphorylation one minute after exposure in a concentration-dependent manner, which resulted in a release of Ca^2+^ from the mitochondria. *Cisplatin* did not produce the same effect even at a concentration of 500 *μ*M [90]. However, another study by Gemba et al. showed that mitochondrial uptake of Ca^2+^ in rat kidney cortical mitochondria was decreased 24 hours after exposure to 500 *μ*M *cisplatin* [91]. 

*Methyl-mercury* (*MeHg*) 0.5–1 *μ*M caused an increase in [Ca^2+^]_i_ *in vitro* in rat cerebellar granule neurons, which decreased cell viability (Table 4). This increase in cell death was prevented 3.5 hours after exposure by using two calcium channel blockers (*ω*-conotoxin and nifedipine) and a calcium chelator (1,2-bis(2-aminophenoxy) ethane-N,N,N9,N9-tetracetic acid tetrakis (acetoxymethyl) ester (BAPTA)). The effect of the calcium channel blockers could indicate that they inhibit the *MeHg* interaction with the target site or block of the entry of *MeHg* in addition to the effects on [Ca^2+^]_i_. BAPTA may have reduced calcium-induced cell death at 3.5 hours after exposure but was unable to prevent *methylmercury*-induced cell death at 24.5 hours. That may indicate that calcium-independent pathways of cell death are involved [92]. The increase of [Ca^2+^]_i_ by *methylmercury* is caused by release from intracellular stores and through an influx of Ca^2+^ into the cell [89].

In HeLa cells, *trimethyl-tin* caused spikes in [Ca^2+^]_i_ as well as sustained increases. The spikes were of variable size and duration and required 0.25 *μ*M *trimethyl tin*. The sustained increase in intracellular calcium was partially reversible and dependent on the concentration of *trimethyl tin* used, where a 5 *μ*M concentration caused an 8% increase in [Ca^2+^]_i_. These effects were independent of external calcium concentrations; however, the increase in [Ca^2+^]_i_ was reduced when the internal calcium stores were compromised [7]. 

Overall, any of the metals affecting any channel or active transport mechanism that involves calcium, at the cellular membrane or the internal stores (as described above) could potentially change [Ca^2+^]_i_.

#### 2.2.4. Calmodulin (CaM)

Calmodulin is a calcium binding protein. Ca^2+^/calmodulin activates CaMK, which phosphorylates synapsin I and opens voltage-activated calcium channels by phosphorylation. Thereby Ca^2+^-influx is increasing which is crucial for releasing the neurotransmitter from vesicles [52, 93].

*Aluminum (Al^3+^)* decreased the biological activity of CaM both *in vitro* and *in vivo *where inhibition *in vivo* is largest in the hippocampus (36.56%), followed by the cerebral cortex (31.76%) and the corpus striatum (22.49%) [20]. *Lead*, however, had an opposite effect as *lead acetate* enhanced CaM activity both *in vitro *and *in vivo* resulting in an increase in CaM-dependent synaptic vesicle protein phosphorylation including the phosphorylation of proteins such as synapsin I. This was proposed as a mechanism for increased spontaneous release of neurotransmitter and depletion of neurotransmitters norepinephrine and acetylcholine following exposure to *lead *[53].

### 2.3. Transporters

#### 2.3.1. Ca^2+^-ATPase

Ca^2+^-ATPase activity in male albino rat synaptic plasma membranes was reversibly inhibited by *Al^3+^* (up to100 *μ*M). This inhibition was concentration dependent with an IC_50_ of 10 *μ*M and resulted in an increase of [Ca^2+^]_i_ [20] (Table 3).

#### 2.3.2. Na^+^/K^+^-ATPase

Cisplatin caused a concentration and time-dependent decrease in Na^+^-K^+^ATPase activity in liver and kidney cells [90]. *Lead* also affected Na^+^-K^+^ATPase activity, and one study showed lowered RBC membrane Na^+^/K^+^-ATPase activity below 60% in 77% of patients with Pb-Rbc ≥ 40 *μ*g/100 mL while only 40% had the same decrease in activity who had a Pb-Rbc ≤ 40 *μ*g/100 mL [94]. *Mercury *compounds*, cadmium, triethyltin, and trimethyl-tin* also inhibit Na^+^-K^+^ATPase activity [95–97]. *Triethyl lead* altered the microviscosity of the plasma membrane of ascites tumor cell and also completely inhibited Na^+^-K^+^-ATPase at concentrations 5–20 *μ*M possibly through direct interaction with its catalytic subunit [98].

#### 2.3.3. Mitochondrial ATPase

Mitochondrial ATPase was inhibited in adult rat brain homogenates with an IC_50_ of 260 *μ*M by *triethyltin* [99]. *Trimethyl tin* has also been shown to affect mitochondrial ATPases *in vitro* [97].

### 2.4. Synaptic Vesicle Associated Proteins

#### 2.4.1. Synaptotagmin I

Synaptotagmin I is a membrane protein, which is hypothesized to be a Ca^2+^-sensor in Ca^2+^-dependent neurotransmitter exocytosis. It has a short intravesicular N-terminus and the cytoplasmic part is composed mostly of two C2 domains, C2A and C2B. The C2A domain is known to bind two Ca^2+^-ions and the binding affinity shows a correlation with the Ca^2+^ dependence of exocytosis [100]. Synaptotagmin I binds phospholipids and syntaxin in a Ca^2+^-dependent manner. The binding to syntaxin is associated with exocytosis. The C2B domain of synaptotagmin I also has Ca^2+^ binding sites and is involved in the Ca^2+^ dependent self-association of synaptotagmin I into multimers [100].

Synaptotagmin I was recently described as a target site for *lead*. Bouton and coworkers suggested a competitive interaction between *Pb^2+^* and Ca^2+^ for the Ca^2+^ binding sites in the C2A domain of synaptotagmin I. At nanomolar concentrations *Pb^2+^* induced the binding of synaptotagmin I to phospholipids with an EC_50 _ of 8 nM. This made it a thousand times more potent than Ca^2+^ at increasing phospholipid binding to synaptotagmin I. Binding of *Pb^2+^* also increased the stability of the secondary structure of synaptotagmin I. A concentration of 2 *μ*M free *Pb^2+^* protected a 32 kDa fragment of synaptotagmin I from proteolytic degradation. It required 11 *μ*M free Ca^2+^ to protect the same size of synaptotagmin I. The same authors showed that *Pb^2+^*, unlike Ca^2+^, did not induce the interaction of synaptotagmin I and syntaxin. Overall, the interaction of *Pb^2+^* was competitive with Ca^2+^ and nanomolar concentrations of *Pb^2+^* could inhibit the ability of micromolar concentrations of Ca^2+^ to induce the interaction of synaptotagmin I and syntaxin [101].

Four binding sites of *Cu^2+^* in the cytoplasmic C2A domains of synaptotagmin I are discussed, three of which are common to Ca^2+^, and one of which is unique to *Cu^2+^*. It was suggested that *Cu^2+^* has a competitive interaction with Ca^2+^, but *Cu^2+^* has a greater affinity for the binding sites common to these metals. Also it was determined that *Cu^2+^* caused a conformational change in the protein, which may make it less susceptible to trypsin cleavage [102]. Kathir and colleagues looked at the interactions between *Cu^2+^* and the C2B domain of p40 synaptotagmin I, which is formed by an alternative translation of the synaptotagmin I gene at the Met103 of the p65 synaptotagmin I. They determined that these interactions stabilized synaptotagmin I bound to phosphatidyl serine vesicles [103].

#### 2.4.2. Synapsin I and p38

Synaptic vesicle associated proteins, synapsin I and p38, in rat CNS decreased on acute exposure of rat to *trimethyl tin*. This decrease was both concentration and time dependent; however, 12 weeks after the exposure, the levels returned to normal. The decline was not a result of loss of tissue which also occurs with *trimethyl tin* exposure but was significantly greater that the reduction in tissue [104].

### 2.5. Neurotransmitters

Release of neurotransmitters is modulated by multiple mechanisms. How metals interfere with some of these pathways has been described above. The following paragraph focuses how metals modulate neurotransmitter levels, their release, and uptake in the presynaptic button.

#### 2.5.1. Effect on Neurotransmitter Metabolism

Treatment of PC-12 dopaminergic neuronal cells with 10 *μ*g/mL *copper* nanoparticles (Ø 90 nm) caused a decrease in dopamine and its metabolites 3.4-dihydroxyphenylacetic acid (DOPAC) and homovanillic acid (HVA). This indicates that the decrease in dopamine may be attributed to a decrease in production and an increase in the breakdown of dopamine [105].

In the same cell line, 10 *μ*g/mL *manganese* nanoparticles (Ø 40 nm) caused a suppression of the tyrosine hydroxylase gene expression, which is involved in the synthesis of dopamine [105]. *Aluminum* has been shown to decrease striatal dopamine content and inhibit the enzyme dopamine-*β*-hydroxylase, which converts dopamine to norepinephrine [106].

Among organic metals, *trimethyl tin* hydroxide treatment of rats on alternate days from days 2–29 of life was shown to decrease the amount of dopamine in the striatum without affecting dopamine metabolites homovanillic acid and dihydroxyphenylacetic acid [107].

#### 2.5.2. Effect on Neurotransmitter Release

*Stannous chloride* increased the amplitude of end-plate potentials in frog neuromuscular junction. A concentration of 10–100 *μ*M *SnCl_2_* increased the quantum of end plate potentials (EPP). However, the miniature end plate potential (MEPP) was not affected. Hattori and Maehashi (1988) suggested that this was due to an increase in the evoked neurotransmitter release while there was no effect on spontaneous release of neurotransmitter. Also, *SnCl_2_* did not increase MEPP amplitude or acetylcholine (Ach) potential, indicating that the sensitivity to ACh was not altered [108]. 

*Lead (Pb^2+^) *in concentrations of at least 100 nM was found to increase the spontaneous release of glutamate and GABA from the presynaptic terminal of rat hippocampal neurons. This effect was found to be concentration dependent and partially reversible and the suggested mechanism of action was through an intracellular signaling pathway [109]. Similarly, it is likely that other metals also affect neurotransmitter release through their interaction with the voltage-gated ion channels, intracellular signaling pathways, and synaptic vesicle associated proteins.

One study looked at the effects of *cadmium* on synaptic transmission by perfusing the amygdala of rats with 10–30 *μ*M* CdCl_2_*. There was an inhibitory effect on the release of excitatory neurotransmitters glutamate and aspartate while the release of inhibitory neurotransmitters glycine and GABA was stimulated [12]. *Aluminum*, as described in previous chapters, affects [Ca^2+^]_i_ and, therefore, as expected, inhibits the release and uptake of GABA from synaptosomes by inhibiting Ca^2+^/calmodulin-dependent calcineurin. It also inhibited pyruvate-supported calcium-evoked acetylcholine release in synaptosomes while in differentiated SN56 cells it decreased acetylcholine release on short-term exposure and increased release on long-term exposure [106].

#### 2.5.3. Effect on Neurotransmitter Reuptake

*Trimethyl tin*, *in vitro,* inhibited the uptake of neurotransmitters GABA, norepinephrine and serotonin, with an IC_50_ of 75, 43, and 24 *μ*M in a concentration-dependent manner in mouse forebrain synaptosomes. *In vivo*, at 2 and 14 hours after *trimethyl tin* exposure, uptake of GABA, and serotonin was decreased whereas there was no significant decline in norepinephrine. These changes in uptake of neurotransmitters could explain their altered levels in the synaptic cleft [110]. However, unlike *trimethyl tin*, *triethyl tin sulfate* had no effect on the levels of dopamine, GABA or acetylcholine in rat brain on exposure for 6 days a week from days 2 to 29 of life in mice [107]. 

*Triethyl lead* caused a concentration-dependent inhibition of Na^+^-dependent high-affinity GABA uptake with an IC_50_ of 10 *μ*M in rat brain synaptosomes. These results were not dependent on Na^+^ and GABA concentration-indicating that competition with Na^+^ and GABA were not the mechanism of action. Triethyl lead also caused a time- and chloride-dependent decrease in ATP [111]. Skilleter showed that *trialkyl lead* at 1 *μ*M causes a decrease in pyruvate uptake by mitochondria in a KCl medium which could possible explain the decline in ATP [112]. However, since the inhibition of GABA uptake occurs before a significant decline in ATP, Seidman and Verity suggested that the inhibition could be due to a defect in GABA binding to uptake site [111]. 

### 2.6. Neurofilaments and Microtubules

*In vivo *exposure of Wistar rats to *arsenic* caused a dose-dependent decrease in neurofilament M and L proteins in the sciatic nerve [113]. These components are required for the formation of a heteropolymer in the cytoskeleton. Since the mRNA expression of these proteins was unaffected, it is possible that the decrease was a consequence of proteolysis. Caplain, which is a calcium-activated cytoplasmic protease, has been implicated in this phenomenon due to the increase in cytoplasmic calcium caused by trivalent *arsenic* [8]. 

*Triethyl lead* also affects microtubules [114–116] and neurofilaments [117]. 50 *μ*M *triethyl lead* caused an inhibition of assembly and a disassembly of microtubules *in vitro* in porcine brain [114]. *Triethyl lead* also caused a reversible perinuclear coil formation of neurofilaments *in vivo* in mouse neuroblastoma cells, which was not associated with a significant change in the microtubules. *In vitro*, *triethyl lead* caused bulging and constriction of isolated neurofilaments from porcine spinal cord, and an unraveling of fibers in preformed filaments [117].

### 2.7. Summary of Presynaptic Effects of Metals

To summarize, presynaptically, voltage-gated sodium, potassium and calcium ion channels are affected by metals such as *Al^3+^, Cd^2+^Pb^2+^, Hg^2+^, cisplatin, Sn^2+^, Zn^2+^, Co^2+^, Ni^2+^, Mn^2+^, Ca^2+^, Mg^2+^, Sr^2+^*, *Ba^2+^,* and * La^3+^*. Mechanisms of effect included binding to a specific target, charge screening, shift of current-voltage curves, and competitive inhibition with the physiological ion or a combination of mechanisms [6, 11, 17, 19, 20, 23–32, 34, 41, 42, 44, 46–50]. Metals also interact with intercellular signaling pathways to modulate synaptic transmission. *Lead* modulated Gs, adenylate cyclase, PKC, and CaM [10, 53, 54, 85]. Adenylate cyclase activity was also modulated by *cadmium, mercury, and zinc * [54, 82, 83] while PKC was inhibited by *lead, aluminum, and nickel,* [20, 69, 85, 86], and IP_3_ was inhibited by *cisplatin and arsenic* [88]. Intracellular calcium was affected through interference with several targets including voltage-gated calcium channels, Ca^2+^ ATPases, and intracellular pathways. *Al^3+^, As_2_O_3_, *and *cisplatin* modulated intracellular calcium [20, 88, 90, 91], and *aluminum,* and *lead* affected Calmodulin activity [20, 53]. Ca^2+^-ATPase activity was inhibited by *aluminum* and Na^+^/K^+^-ATPase activity was modulated by *lead, cisplatin, mercury,* and *cadmium* [20, 90, 94–96]. Synaptotagmin I, a synaptic vesicle associated protein, was a target site for interaction with *Pb^2+^* and *Cu^2+^* [101–103]. Neurotransmitter release was possibly affected through interaction with many of the targets above as well as through interaction with synthesis and degradation of neurotransmitters and enzymes in the metabolic pathway, which resulted in modulation of neurotransmitter release by metals such as *copper, manganese,* and *tin* [101–103]. The mRNA expression of neurofilaments was affected by *arsenic* [113]. Often contradictory results were obtained regarding the effects of metals, which may indicate that metals had different effects on targets depending on the state of the metal, its concentration, the medium, the area of the brain, and whether the experiment was *in vivo *or *in vitro*.

## 3. Postsynaptic Targets 

The activation of ligand-gated receptor channels is vital for controlling nerve cell inhibition or excitation and, therefore, fashioning the response of individual neurons, neuronal networks, and, ultimately, the entire brain. Consequently the resulting currents through the associated channels will either depolarize or hyperpolarize the postsynaptic terminal under different physiological conditions. The major *excitatory* neurotransmitter in the brain is L-glutamate. There are three classes of ionotropic glutamate receptors named according to their potent excitatory amino acids: *α*-amino-3-hydroxy-5-methyl-4-isoxazolepropionic acid (AMPA), kainate, and *N*-methyl-D-aspartate (NMDA). The AMPA and kainite-activated channels are designated non-NMDA receptor-channels and will be further discussed in Section 3.2 while metal actions at the NMDA/receptor channel complex are analyzed in the upcoming Section 3.1. 

**Figure 4:**
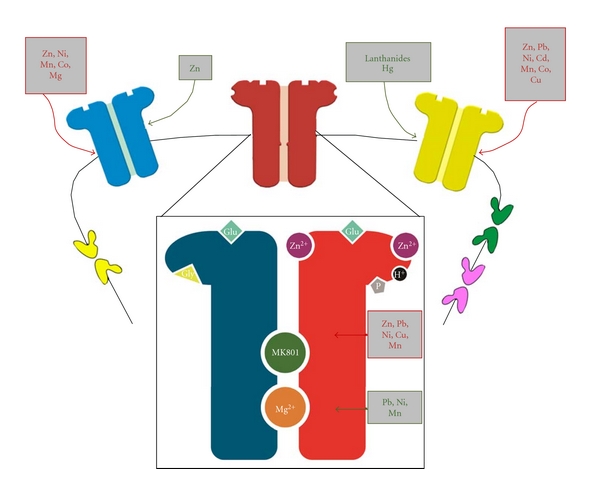
Postsynaptic ligand-gated ion channels as targets of neurotoxic metals. The main postsynaptic channels are the AMPA/kainate and NMDA receptors whereas the main inhibitory receptors are the GABA_A_Rs. Each receptor represents a target for multiple metals. The NMDAR has many modulatory sites identified as it is more extensively studied. NMDAR is composed of a heteromer made of NR1 and NR2, each having multiple subtypes. In the diagram blue arm represents NR1 while the red arm represents NR2, the main modulatory subunit. Most metals have been shown to have effects on NR2 subunit (for values regarding the specific subtypes refer to Section 3.1 and Table 5).

The most abundant *inhibitory* neurotransmitter in the brain is **γ**-aminobutyric acid (GABA), which acts on its own class of ligand-gated channels. However, these receptors are subject to modulation by other compounds and ions, including metals [118] (Figure 4). As mentioned in the introduction, biological systems utilize metals because of their catalytic versatility, but the high affinity of these metals to specific binding sites could possibly severely impair synaptic transmission and, therefore, cause a malfunction of neuronal networks which might result in changes in perception, learning and memory, and finally change behavior, even at very low and environmentally relevant concentrations.

### 3.1. The NMDA-Receptor/Channel-Complex

The *N*-methyl-D-aspartate receptor (NMDAR) is a subtype of glutamate ionotropic receptors. The most widely distributed and studied NMDARs are tetrameric assemblies composed of two NR1 subunits and two of the four different NR2 types (named A, B, C, and D), of which NR2A and NR2B are most common [119, 120]. The physiological and pharmacological properties of these receptors are dependent on the NR2 subunit, although different NR1 splice variants may also influence channel performance [121, 122]. NMDAR subunits have a characteristic modular architecture consisting of two extracellular domains, the regulatory amino terminal domain (ATD) and the agonist-binding domain (ABD), and three membrane-spanning segments (M1, M3, and M4) and a reentrant hairpin-like pore loop, M2 [123]. 

The associated NMDAR channel requires simultaneous binding of two agonists, glutamate (Glu) and glycine (Gly), for opening (for review, [124]). Gly has its binding site in the ABD region of NR1 whereas NR2 ABD binds Glu [120]. The receptor-channel complex has unique properties such as a high Ca^2+^ permeability. Also, the functional activation of NMDAR channels is linked to a voltage-dependent *magnesium-* (*Mg-*) mediated block [125, 126]. Extracellular *Mg^2+^* inhibits NMDA responses at membrane potentials close to the resting membrane potential [127]. Studies of the site of action of *Mg^2+^* reveal that the N and N+1 site on NR2 subunit are important for the *Mg^2+^* block [128]. When the membrane potential is sufficiently depolarized, *Mg^2+^* leaves its binding site and even potentiates NMDA responses in low glycine concentrations. This potentiation is shown to be due to increased NMDAR affinity to glycine, in all neurons [129]. However, there was also glycine-independent potentiating effect of *Mg^2+^*, which appeared to be largely voltage-independent and subunit specific, being seen only with NR2B-containing receptors. This potentiation has an EC_50_ of *∼*2 mM [130]. 

All of these effects reveal the complicated modulation by *Mg^2+^* on NMDAR currents. Some data suggest that *Mg^2+^* and spermine may completely or partially share a common binding site; similar observations are obtained using spermine [130, 131]. Three different steps in the action of these two substances could be distinguished: (1) increase in glycine affinity, seen in all neurons; (2) voltage-dependent block, also seen in all neurons; and (3) glycine-independent potentiation that was subunit specific [132, 133]. 

NMDARs contain a number of distinct recognition sites for other endogenous and exogenous ligands, which modulate their functions, such as divalent metal cations, as explored in the later sections (Figure 4). 

*Zinc* is the second most prevalent trace element in the body. Most of the *zinc ions (Zn^2+^)* are trapped within proteins, but some of it is loosely bound (chelatable zinc) [134]. In the mammalian brain, chelatable *zinc* is distributed mainly in the forebrain and localized almost exclusively within synaptic vesicles of a subset of glutamatergic axon terminals [135]. Since it is accumulated in synaptic vesicles, it has been assumed that *zinc *is released, with glutamate, during neuronal activity. Many studies have showed evidence of quantal corelease of *zinc* and glutamate (for review, [134]). 

NMDARs are the best characterized synaptic zinc targets. At low micromolar concentrations, *Zn^2+^* selectively inhibited NMDAR-mediated responses. The major effect was through voltage-independent, noncompetitive inhibition seen as a decrease in the opening probability of the channel [136–138]. However, at concentrations higher than 20 *μ*M, *Zn^2+^* could also produce voltage-dependent inhibition, probably by binding inside the pore at the *Mg^2+^* blocking site [139]. It had been proposed that *zinc* is an endogenous ligand controlling NMDARs functions [140]. 

An important consideration in NMDAR function and pharmacology is that the *Zn^2+^* binding to NR2A and NR2B subunits is associated with discrete subunit selectivity [141]. NMDARs containing the NR2A subunit had a very high sensitivity to extracellular *Zn^2+^* (IC_50_ *∼*15 nM) [133, 142]; however, this inhibition never exceeded 60–80% [142]. The mechanisms of this inhibition occurred in different steps [143]: in the first step *Zn^2+^* bound in the interlobe cleft of the NR2A-NTD promoting its closure, which would exert tension on the linkers connecting NTDs to ABDs. This effect would secondarily cause a disruption of the ABD dimer interface. In turn, this disruption relieved the strain on the transmembrane segments, and with proton binding, it allows the closure of the channel gate [133]. This mechanism of enhancement of proton inhibition was supported by subsequent work [144]. 

*Zinc* has a much lower affinity to the NR2B subunit, compared to NR2A, with voltage-independent inhibition (IC_50_ *∼*1 *μ*M) [122, 145]. It was suggested that the mechanism of inhibition might be similar to the mechanism described for NR2A receptors. However, *zinc* inhibition of NR2B receptors appeared to not be dependent on pH [122], suggesting that this inhibition might occur through a different mechanism [133]. Studies showed that *Zn^2+^* bound with high affinity to a site in NR2A ATD region [146] and with a lower affinity to a site in the same region of the NR2B [145]. The affinities of NR2C and NR2D to *Zn^2+^* described to be even higher (IC_50_> 10 *μ*M) [134]. 

*Lead (Pb^2+^)* is an exogenous heavy metal, which has been a public health concern due to its widespread contamination and its multiple toxic effects. Effects of acute exposure to *Pb^2+^* in the micromolar range were originally described in cultured and acutely dissociated neurons as a reversible inhibition of the NMDAR current [19, 147–150]. These studies outlined several features of the effects of *Pb^2+^* (for details, see review [151]). First, the inhibition was specific for NMDA channels, which were significantly more sensitive to *Pb^2+^* inhibition than other glutamate channels. Secondly, the channel block was independent of voltage [148–150], and therefore the interaction site was likely to be located away from the electric field, or outside the conducting pore. Thirdly, the effect was noncompetitive since increasing the glutamate or glycine concentration could not overcome the block of the current [148, 152]. Biochemical studies suggested that the inhibitory effects of *Pb^2+^* on NMDA receptors were age- and brain-region specific [152–154]. One important observation in *Pb^2+^* neurotoxicity was that the hippocampus appears to be more sensitive than other brain regions [153, 155, 156]. 

The effect of *Pb^2+^* on glutamate and NMDA-evoked currents depended on the subunit composition of the receptor-channel complex. Concentration-dependent *Pb^2+^* inhibited the currents activated by either Glu or NMDA in oocytes expressing NR1-2A or NR1-2B (Table 1, [157]). Yamada and colleagues [158], however, showed that higher concentrations were needed than mentioned before, although, there were methodological differences between the two studies, which could account for the different results (for details, [157]). 

Also, *Pb^2+^* at low concentrations (<1 *μ*M) acted as a positive modulator of agonist action on NR1-2AB and NR1-2AC receptors whereas at higher concentration *lead *inhibited NR1-2AB and NR1-2AC receptors, but with less potency compared to NR1-2A or NR1-2B [157, 159].

There is incongruity whether *lead* acts via the *zinc* binding site or through a different site. A set of experiments demonstrated that in the presence of increasing amounts of *Pb^2+^*, there was a concentration-dependent downward shift of the *Zn^2+^* inhibition curve; also, the values of IC_50_ for *Zn^2+^* inhibition decreased as a function of increasing *Pb^2+^* concentrations. The effects of *Zn^2+^* on *Pb^2+^* curve and IC_50_ were analogous [160]. These findings suggested that the two metals act via independent binding sites, which is in line with the observation that increasing concentrations of *Pb^2+^* did not affect the *Zn^2+^* IC_50_ [154]. However, these results were in contrast with other findings which report that the two cations compete for the same binding site [161, 162]. 

*Nickel (Ni^2+^)* is a trace element, which is essential for many biological organisms, but could also induce toxicity. The effects of *Ni^2+^* on NMDA channel activity were described as a voltage-dependent and “*Mg^2+^*-like” inhibition [127]. Later work showed a potentiation of homomeric NR1a channels [121] and an inhibition of NR1-2A channels [13, 146]. In more recent studies, it is suggested that, besides the voltage-dependent *Mg^2+^*-like inhibition, *Ni^2+^* causes a potentiation of NR2B-containing channels and a voltage-independent inhibition (*Zn^2+^*-like inhibition) in those neurons containing NR2A [163]. 

*Nickel* also caused a reduction of single channel current amplitude at negative voltages while the dependence on membrane voltage was slightly steeper for NR2A than NR2B [13]. Several analogies with *Mg^2+^*-like inhibition indicated that it might interact with either the N or N+1 site in the pore-forming region of the NR2 subunit [13]. Support for the above theory came from an experiment showing that a single mutation in the NR2B site at the N+1 site would completely abolish the voltage-dependent block *Ni^2+^* [123]. The N+1 residue had been shown to be a critical binding site for *Mg^2+^* block in NR2A subunit [128]. Moreover, at positive potentials the effects of *Ni^2+^* were highly subunit dependent. NR2A-containing channels were blocked in a voltage-independent manner whereas NR2B containing channels were facilitated [13] (see Abbreviation section). However, at higher concentrations (IC_50 _= 442 *μ*M), a voltage-independent inhibition was also present in NR1-2B channels [123]. The voltage-independent inhibition site of *Zn^2+^* was investigated as a potential site for *Ni^2+^* inhibition, but this did not seem to be the case. Besides the difference in blocking affinity, *Zn^2+^* inhibition was pH dependent [122, 164] while *Ni^2+^* inhibition was not [163]. Also*, Zn^2+^* inhibition was never more than 60–80% (as described in section  3.2.1), while *Ni^2+^* inhibition approached 100% at positive potentials [123]. Other results also showed that mutations that affect the inhibition of *Zn^2+^* did not modify *Ni^2+^* sensitivity [146]. 

The NR2B-selective potentiation was suggested to share the site of action with spermine, as *Ni^2+^* partially obscured the effect of spermine when they were applied concurrently [123]. 

Although the actions of *zinc, lead, and nickel* on NMDARs were intensively investigated, there are also some reports that other metals have an effect on these receptors and channel activity. 

*Copper (Cu^2+^)* is an endogenous metal in the human brain [165], and it is an established fact that *copper* represents an integral part of neurotransmission [166]. It is released from synaptic vesicles following neuronal depolarization [167]. The concentration of *copper* in the synaptic cleft could reach up to 100 *μ*M [168]; later studies estimated the concentration of *copper* released into the synaptic cleft to be in the range of ~15 *μ*M [169, 170]. However, the topographic distribution showed marked variations between different brain areas [171]; the highest concentration of *copper* has been found to be in the hypothalamus [172]. 

*Cu^2+^* acts on NMDA receptors and reduces the current—induced by 50 *μ*M NMDA—in a concentration-dependent manner with an IC_50_ of 15.9 *μ*M. This block was completely and quickly reversible, even in the absence of antioxidant dithiotreitol, suggesting that the inhibition was not an oxidizing effect [173]. Further studies showed that *Cu^2+^* inhibition was characterized by voltage-independent, but use-dependent mechanism of action, as the degree of inhibition was dramatically decreased in the absence of agonists [174]. 

Another trace metal required for normal brain function is *manganese (Mn^2+^)*. In the human brain*, Mn^2+^* is most concentrated in the globus pallidus, caudate, and putamen, but also found in other areas [175]. *Mn^2+^* produces a strong voltage-dependent block in response to NMDA [176]. It was, also, a competitive antagonist of MK-801 binding to the NMDAR-channel. Its inhibitory effects were activity-dependent since *Mn^2+^* was a more potent inhibitor in the presence of NMDA coagonists (Glu and Gly) than in their absence [177]. Taking these studies together, they indicate that *Mn^2+^* is an NMDAR channel blocker. Interestingly, the inhibitory constant for *Mn^2+^,* in the absence of Glu and Gly, was significantly different in neuronal membranes from the cerebellum relative to other brain regions; however, in the presence of the agonists, *Mn^2+^* was equally potent in inhibiting NMDARs in different brain regions [177]. 

#### 3.1.1. The Glycine-Binding Site of the NMDA-Receptor/Channel Complex

The NMDAR glycine-binding site was susceptible to modulation by divalent cations, especially when the glycine site was not saturated. Low, extracellular concentrations of *Mg^2+^* potentiated NMDAR currents. The potentiation was the result of an increase in the affinity of NMDAR for glycine [129, 130, 178]. The mean glycine EC_50_ value was 100–133 nM in control conditions and was reduced to 60–62 nM in the presence of 10 *μ*M *Mg^2+^* [178]. This increase in affinity was also demonstrated by decreasing the inhibitory potency of NMDAR glycine-site antagonists upon the addition of potentiating concentrations of *Mg^2+^*[129, 178, 179]. Ca^2+^ had the same effect as *Mg^2+^* [129, 178, 179]. 

Concentrations of *Pb^2+^* and *Zn^2+^* higher than 10 *μ*M inhibited NMDAR potentiation by Ca^2+^ and *Mg^2+^* [178]. These findings were supported by other studies, which showed that increasing concentrations of Ca^2+^ diminished the inhibition of NMDAR currents by *Zn^2+^* [136], or *Pb^2+^* [180]. It is suggested that these divalent cations act on the same site, and Ca^2+^ and *Mg^2+^* have opposite effects on glycine binding compared to *Pb^2+^* and *Zn^2+^*  [178]. 

Even the presence or absence of glycine modulated the effects of some of the cations: for example, *Cu^2+^* and *Mn^2+^* are both potent NMDAR channel inhibitors (as shown above), but in the presence of glycine and glutamate, *Cu^2+^* was more potent than *Mn^2+^*, and in the absence of glycine, *Mn^2+^* was slightly more potent [177]. 

### 3.2. AMPA and Kainate Receptors

The two classes of ionotropic glutamate receptor-channels, which are designated non-NMDA channels, are AMPA (*α*-amino-3-hydroxy-5-methyl-4-isoxazolepropionic acid) and kainate receptors, named after their most potent excitatory amino acids. The AMPAR channel is also activated by kainate (for review, [181]). Also, AMPARs mediate the fast excitatory synaptic transmission in the CNS [182]. 

AMPA/kainate receptor-gated channels are permeable to Na^+^ and K^+^ and more or less impermeable to Ca^2+^; however, there is a subpopulation of central neurons, which contain AMPA/kainate receptors with enhanced Ca^2+^-permeability [183, 184]. This Ca^2+^ conductance triggered by the AMPA/kainate receptors seemed to be dependent on the absence of the GluR2 subunit [119, 182, 185]. 

Extracellular calcium ions produced rapid and reversible voltage-independent inhibition of AMPARs, with both Ca^2+^ permeable and Ca^2+^ impermeable AMPAR being equally sensitive [186]. The Ca^2+^ effects were agonist dependent, more prominent in the case of AMPA compared to Glu or kainate. These data suggested that Ca^2+^ enhances desensitization, as two well-known antidesensitization agents prevented Ca^2+^ inhibition through Ca^2+^ binding to a modulatory site in the AMPAR [186]. 

Effects of *zinc* on AMPA/kainate receptors have also been explored. *Zn^2+^* appeared to have a dual effect on AMPAR: at micromolar concentrations, it enhances AMPA receptor responses whereas at millimolar concentrations, it inhibits them [136, 187]. These effects of *Zn^2+^* appear to be subunit specific as well. Experiments using cloned AMPAR expressed in oocytes demonstrate that, in normal calcium-containing solution, *zinc* could potentiate current from homomeric GluR3 receptors over a narrow range of 4–7.5 *μ*M *Zn^2+^* while homomeric GluR1 receptors could not be potentiated, but are inhibited by 10 *μ*M *Zn^2+^* [188]. Additionally, in calcium-free solution, the inhibition caused by *Zn^2+^* on GluR1 shifted to ≥1 mM and potentiation was attainable reaching a maximum of *∼*200% at 50 *μ*M *Zn^2+^*. Also, GluR3 showed maximum potentiation not significantly different from GluR1 potentiation. The presence of GluR2 subunit in heteromeric expression of GluR2/GluR3 prevented the potentiation by *Zn^2+^*, but also allowed inhibition (with 500 *μ*M *Zn^2+^*, current was 39% of control). The presence of GluR2 rendered the effects of *Zn^2+^* independent of Ca^2+^ levels (for details, [189]). 

The effects of other divalent metals effects were explored on these channels but less extensively compared to NMDARs. Various metals caused inhibition of Ca^2+^ impermeable AMPAR with the following rank order of inhibition: *Ni^2+^*>* Zn^2+^*>* Co^2+^*>* Ca^2+^*>* Mn^2+^*>* Mg^2+^* (for values, refer to Table 5) [190]. The proposed mechanism of action is that complexes of divalent cations and AMPAR agonists compete with the free agonists rather than the cations themselves. This mechanism fits the data in which a competitive type of inhibition is observed; in addition, an increase in agonist concentration reduce the inhibitory effects of divalent metals less than that of DNQX (the classical competitive AMPAR antagonist) [190]. 

### 3.3. GABA Receptor

**γ**-Aminobutyric acid (GABA) is the major inhibitory neurotransmitter in the mammalian central nervous system. The synaptic transmission mediated by GABA_A_ receptor-channel complex leads to a hyperpolarization of the cell membrane due to the fast activation of postsynaptic chloride channels upon the exposure to GABA [191]. The GABA_A_R is comprised of pentameric combination of *α*1–6, *β*1–4, *γ*1–3, *δ* 1, and/or *ε*1 subunit subtypes that form an intrinsic chloride ion channel, and each subunit comprises four domains. GABA_A_Rs have some recognized allosteric binding sites such as barbiturates, benzodiazepines, and picrotoxin [192, 193]. The properties of the allosteric binding sites were influenced by the subunit subtype composition of GABA_A_R (for review [194]). 

#### 3.3.1. Inhibitory Effects of Metal Ions

The GABA_A_R response to GABA-evoked currents was modulated by a number of divalent metal cations. *Zinc, cadmium, nickel, manganese, cobalt, lead, *and* copper* inhibited the response to GABA. The inhibition by divalent metals has consistently been shown to be reversible with no or little voltage dependence [14]. 

*Zn^2+^* had the potential to directly interact with the GABA_A_R to influence inhibitory postsynaptic currents (IPSC) amplitudes and kinetics [118]. *Zn^2+^* suppressed the GABA-induced chloride current with a *K*_d_ of 19 *μ*M in a noncompetitive, voltage-independent manner, and without interference with any of the allosteric sites on the GABA-R [195]. Using cultured hippocampal neurons, studies showed that *Zn^2+^* reduced the amplitude, slowed the rise time, and accelerated the decay of mIPSCs. Evidence indicated that inhibition of mIPSCs by *Zn^2+^* was attributed to an allosteric modulatory site located on the extracellular domain of GABA_A_ receptors [196, 197]. In accordance with the previous hypothesis, single-channel studies have also shown that *Zn^2+^* reduced the opening frequency with no evidence of flickering [198–200]. 

From another perspective, the effects of *Zn^2+^* were subtype-specific. The *zinc-*sensitivity of the channels seemed to be dependent on the absence of *γ* subunits, as its presence in any combination with other subunits led to the formation of GABA_A_ receptors almost insensitive to *Zn^2+^* [201, 202]. Furthermore, the presence of a *δ* subunit enhanced *zinc* sensitivity [202, 203]. The exchange of a particular subunit with other members of the same subunit family (*α*1 versus *α*3, *β*1 versus *β*2, and *γ*1 versus *γ*2) did not alter the large difference in *Zn^2+^* sensitivity between GABA_A_R containing or lacking *γ* subunit [202]. Later studies showed that recombinant GABA_A_R, which contain *α*4, *α*5, and *α*6 subunits, were more sensitive to *zinc* than those that contain *α*1 subunits [204–207]. Given that the majority of synaptic GABA_A_R are of the *α*-, *β*-, *γ*-isoform [208], together with the above studies, indicate that a likely target of *Zn^2+^* modulation is an extrasynaptic *α*-, *β*- or *δ*-receptor [118]. 

In rats dorsal root ganglion (DRG) neurons, *Cu^2+^* at concentration of 15 *μ*M, suppressed the peak amplitude of the GABA-induced current to approximately 50%; the blocking was exerted and reversed quickly, and it was independent of membrane potential [195].

The similar blocking profiles of *Cu^2+^* and *Zn^2+^* led to the question whether they shared a common binding site. Competition experiments showed that *Zn^2+^* suppression of GABA-induced current was decreased with increasing concentrations of *Cu^2+^*, suggesting that *Zn^2+^* and *Cu^2+^* act on the same allosteric site to inhibit GABA_A_R [55]. 

In a later study, the *copper*-induced block of GABA_A_R in Purkinje cells developed slowly, was poorly reversible, and decreased with increasing GABA concentrations. The block occurred at low concentrations indicating a high affinity with an IC_50 _~35 nM [209]. The copper block of GABA_A_R in Purkinje cells seemed to have a higher affinity compared to the block in DRG [195] and olfactory bulb neurons [210]. Another difference between these tissues was that *Cu^2+^* in DRG cells interacts in a noncompetitive manner while in Purkinje, *Cu^2+^* decreased the potency of GABA without affecting the maximal response. A possible explanation for this discrepancy might be different subunit composition of the GABA_A_R [210]. 

The effects of multiple divalent metals (*cadmium, nickel, manganese, zinc,* and *barium*) were shown in a study of GABA responses of embryonic chick spinal cord neurons. The results were suggestive of an allosteric mechanism of inhibition of GABA_A_R currents. Through combination experiments they showed that the ions acted at a common site but possessed different intrinsic efficacies with the following rank: *Zn *>* Cd*>* Ni*>* Mn*. *Ba* was thought to bind to the site but lacked efficacy as an inhibitor of the GABA response [14]. The rank of efficacy was supported by other experiments [195]. 

#### 3.3.2. Excitatory Effects of Metal Ions

*Lanthanides* comprise a series of 15 metals starting with *lanthanum (La)* and ending with *lutetium*. In sub-millimolar concentrations, lanthanum ions modulate GABA-induced currents [55, 191, 211]. 

*La^3+^* increased the affinity of GABA for the receptor in a concentration-dependent manner with an EC_50_ = 231 *μ*M and maximum enhancement to about 300% of control with 1 mM. This potentiation was completely and quickly reversed, but was more enhanced as the potential became more negative (1.6% per 10 mV) [195]. Also, this effect was independent of the presence or absence of barbiturates, benzodiazepines, picrotoxin, or *Zn^2+^/Cu^2+^*, indicating that it was bound to a site different from all of the binding sites of the above substances [55]. *La^3+^* did not activate transmembrane currents, it only potentiated GABA-induced currents; also *La^3+^* did not affect the amplitude of the maximum response induced by GABA. These data together suggested that *La^3+^* increased the affinity of GABA_A_R to its agonist [212]. *Other lanthanides* exhibited enhancing actions, and the efficacy increased with increasing the atomic number, such that *Lu^3+^* (1 mM) increased the current to 1230% of control [55]. However, previously it was reported that *lanthanides* generate inward currents on their own in the absence of GABA [55]. This controversy might be due to use of different tissues; as *La^3+^* did not activate transmembrane currents in CA1 hippocampal pyramidal neurons whereas *La^3+^* generated inward currents in DRG neurons. 

Additionally, recombinant GABA_A_R studies suggested that changing the *α*-subunit subtype from *α*1 to *α*6 alters the effects of *lanthanum* from potentiation to inhibition at comparable concentrations. These studies also suggested that the maximal inhibition of GABA_A_R current by *La^3+^* in *α*6-containing receptors is greater in the presence of *δ* subunit (83%) than in the presence of *γ* subunit (32%) [213].

Another cation that might affect GABA_A_R is *mercury (Hg)*. In its inorganic form, GABA_A_R channel complex was strongly stimulated by low concentrations of *Hg* [214]. *Mercuric chloride* (100 *μ*M) increased the GABA-induced current to 270% of control, and increased it to 115% of control with 0.1 *μ*M [55] indicating its high potency.

### 3.4. Summary of Postsynaptic Effects

The main **postsynaptic targets** are the ligand-gated receptors including, but not limited to, NMDA, AMPA/kainite, and GABA receptors. Of those, NMDAR channels are the most widely studied receptors due to their association with disease status. Relatively fewer studies have been done on other targets, which could lead to underestimation of their roles in metal toxicity. 

Two main mechanisms established for metals effects on NMDAR: Mg^2+^-like inhibition, which is voltage dependent, or Zn^2+^-like inhibition, which is voltage independent. *Lead* and *copper* were found to inhibit NMDAR in Zn^2+^-like pattern. However, *copper*-mediated inhibition of NMDAR was use dependent which was also true for *manganese-*mediated inhibition [160–162]. *Nickel *on the other hand, showed an Mg^2+^-like inhibition at negative potentials. However, it had different effects on NR2B and NR2A containing channels at positive potentials. It caused a potentiation of NR2B-containing channels and a Zn^2+^-like inhibition in those containing NR2A. However, at high concentrations, NR2B-containing receptors also showed Zn^2+^-like inhibition at positive potentials [163]. Another major target on the NMDAR was glycine-binding site with multiple metals affecting it. Ca^2+^ and Mg^2+^ potentiated NMDAR currents whereas *Pb^2+^* and *Zn^2+^* inhibited NMDAR currents. All of these four divalent metals might act on the same binding site with different effects [178]. Also, the presence or absence of glycine affected the potency of *Cu^2+^* and *Mn^2+^*, such that in the presence of Gly, *Cu^2+^* was more potent, whereas in the absence of Gly, *Mn^2+^* was slightly more potent [177].

The main inhibitory receptors in the brain, GABA_A_, are also modulated by a variety of metals. Certain metals suppressed the GABA-induced chloride current while others augmented it. Multiple divalent metals had inhibitory effects on GABA-induced current. *Cu^2+^* and *Zn^2+^* suppressed GABA_A_R in an equipotent manner, and there was some evidence that they act on the same site. Other divalent metals also showed inhibitory effects through an allosteric mechanism of inhibition, and they demonstrated a common site of action but with different intrinsic efficacies with the rank *Zn^2+^*>* Cd^2+^*>* Ni^2+^*>*Mn^2+^* [14].

Interestingly, *Lanthanides* exhibited enhancing effects of GABA-induced currents. The effect was completely and quickly reversible. The efficacy increased with increasing atomic number [55]. However, recombinant GABA_A_R studies suggested that changing the *α*-subunit subtype could alter the effects of lanthanum from potentiation to inhibition in a comparable concentration range.

*Mercury* was another metal, which had inhibitory and excitatory effects. In its inorganic form, GABA_A_R channel complex was strongly stimulated by low concentrations of *Hg*. However, *methyl mercury* was a potent inhibitor of GABA-induced current, and this effect was irreversible [55]. This showed how organic metals might behave differently compared to inorganic cations; however, there is less information in the literature about the effects of organic metals. One of the most toxic organic metal is *trimethyl-tin* (*TMT*). At a concentration of 100 *μ*M, around 20–30% of AMPAR and GABA_A_R currents were inhibited whereas 35% of NMDAR ion currents were blocked [215].

## 4. Disruption of Synaptic Plasticity

Multiple reports have demonstrated that human exposure to environmentally concentrations of certain metals can result in cognitive deficits. 

*Arsenic* consumption, mainly through contaminated water, has been found to be associated with impairment of long-term memory and a reduction in the verbal IQ of children [216, 217]. *Lead *has been studied extensively for its role in disruption of synaptic plasticity in an attempt to explain the cognitive deficits observed in children with elevated blood lead levels. The CDC currently considers blood Pb^2+^ level of 10 *μ*g/dL to be the threshold for impairment of cognitive function in children [218], although recent studies have observed that cognitive impairment can occur even at blood lead levels <10 *μ*g/dL [219]. There have been reports that *aluminum* also affects synaptic plasticity, which has been implicated in the pathogenesis of Alzheimer's disease, although this topic is highly debated. It has been argued that these detrimental effects on learning, memory, and cognition, which are associated with exposure to metals, may be linked to the disruption of processes that are involved in synaptic plasticity. The formation of long-term potentiation (LTP) is impaired in mice that have inborn low learning capacity indicating the crucial role for synaptic plasticity as the basis of learning and memory. Impairment of LTP has been observed with exposure to *lead *[220]. Moreover, studies have shown that *aluminum* also impaired hippocampal long-term potentiation (LTP) and long-term depression (LTD) in rats both *in vivo* and *in vitro* [221, 222]. Also, multiple metals have been shown to have different concentrations in patients with Parkinson's disease compared to healthy individuals, and the levels of aluminum have been identified as a potential diagnostic marker [223].

To understand how metals and their compounds affect learning and memory, their effects on different stages of LTP and LTD were compared to identify specific sites of interaction for particular metals as well as targets common to more than one metal. 

**Figure 5:**
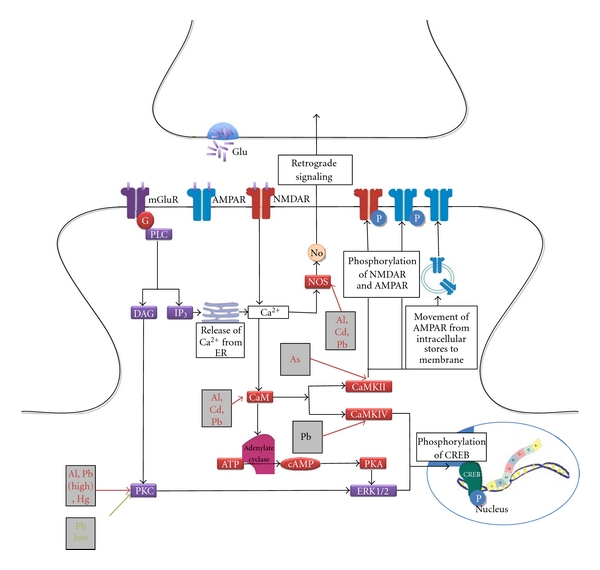
Proteins involved in the formation of long-term potentiation (LTP) and toxic effects of metals. LTP consists of different forms: early-phase LTP (E-LTP), which lasts only a few hours, and late-phase LTP (L-LTP), which lasts for several days. E-LTP includes short-term potentiation (STP), which is dependent on NMDA receptor activation and Ca^2+^/calmodulin and LTP-1 that involves protein kinase C (PKC) and Ca^2+^/calmodulin-dependent protein kinase- (CaMK-) dependent phosphorylation. While STP can be formed by activation of NMDA and calmodulin dependent enzymes, LTP-1 requires activation of PKC via DAG that is produced after the activation of mGluRs. PKC and CaMKII then phosphorylate AMPA and NMDA receptors. L-LTP consists of the later phases of LTP, which are LTP-2 and LTP-3. LTP-2 requires synthesis of new proteins and receptors whereas LTP3 requires gene transcription. Activation of adenylate cyclase and cAMP-dependent activation of PKA are required for the formation of the later phases of LTP. LTP-3 depends on the activation of extracellular signal- related kinase 1/2 (ERK1/2) and CaM kinase IV, which in turn phosphorylate CREB and lead to new protein synthesis. Other factors such as p38 mitogen-activated protein kinase (p38 MAPK) leads to the formation of long-term depression (LTD). Several of the molecules required to produce these different forms of LTP have been identified and are targets for metal toxicity, which have been shown (red arrows indicate inhibition whereas green arrows indicate activation by metals. Black arrows indicate activation that occurs during normal formation of LTP).

Recently, it has been shown that LTP consists of different succeeding forms: early-phase LTP (E-LTP), which lasts only a few hours, and late-phase LTP (L-LTP), which lasts for several days [224–226]. Several of the molecules required to produce these different forms of LTP have been identified and are targets for metal toxicity [224, 225, 227] (refer to Figure 5 and Table 6).

**Early-LTP**

it includes short-term potentiation (STP), which is dependent on NMDA receptor activation Ca^2+^/calmodulin; and LTP-1 that involves protein kinase C (PKC) and Ca^2+^/calmodulin-dependent protein kinase- (CaMK-) dependent phosphorylation. While STP can be formed by activation of NMDA and calmodulin-dependent enzymes, LTP-1 requires activation of PKC via DAG that is produced after the activation of mGluRs. The activity of mGluR-PKC is important for both increasing activity as well as increasing number of AMPA receptors. PKC and CaMKII then phosphorylate AMPA and NMDA receptors.

**Late-LTP**

there are two later phases of LTP named LTP-2 and LTP-3. LTP-2 requires synthesis of new proteins and receptors whereas LTP-3 requires gene transcription. Activation of adenylate cyclase and cAMP-dependent activation of PKA are required for the formation of the later phases of LTP. LTP-3 depends on the activation of extracellular signal-related kinase 1/2 (ERK1/2) and CaM kinase IV, which in turn phosphorylate CREB, and this leads to new protein synthesis. p38 mitogen-activated protein kinase (p38 MAPK) is involved in the formation of long-term depression (LTD), and c-JUN-N-terminal kinase (JNK) is thought to participate in LTD [228, 229].

### 4.1. Disruption of Long-Term Potentiation by Exposure to Metals in Adults

#### 4.1.1. Calmodulin

Calmodulin (CaM) is a regulatory protein that is activated by [Ca^2+^]_i_. This protein is found in high concentrations in CNS neurons and is involved in the activation of several other proteins. Some of the CaM-regulated proteins that modulate synaptic plasticity include adenylyl cyclases (AC1 and AC8), protein kinases, calcineurin, calmodulin kinases (CAMK I, II, and IV), nitric oxide synthase, and Ca^2+^ conducting channels. CaM has four Ca^2+^ binding sites. Ca^2+^ binding to CaM leads to a conformational change that exposes a hydrophobic domain which enhances the binding of CaM to other target proteins [226]. It has been hypothesized that in absence of Ca^2+^, the concentration of free CaM is regulated by neurogranin that binds CaM and releases free CaM in response to PKC and Ca^2+^ [226, 230, 231]. This important molecule has been identified as a target of several neurotoxic metals such as *aluminum, cadmium, *and *lead*.

*CaM, *when incubated with increasing concentrations of* aluminum (Al^3+^)* (from 0–1000 *μ*M), showed decreased activity. This decrease in activity, measured by the ability of CaM stimulate activator-deficient cAMP phosphodiesterase was concentration dependent, [20]. Yet, another study showed that an [*Al^3+^*]: [CaM] ratio of 3 : 1 resulted in 50% decrease in phosphodiesterase activity, and maximal inhibition was observed at a ratio of 4 : 1 [60]. 

Recently, using highly specific monoclonal antibodies that detect the different conformational states of CaM and monoclonal antibodies against *Al*-CaM complex, researchers found that on dissolving CaM with *AlCl_3_* **·**6H_2_O (in increasing concentrations from 0–480 *μ*M), the antibody specific to Ca^2+^ calmodulin conformation (the active form) mAb CAM-1, did not recognize the *Al*-CaM complex (at *Al* concentrations of 240–300 *μ*M) indicating that the CaM was in the inactive conformation. Moreover, the antibodies against the *Al*-calmodulin complex were found to bind to their antigen in the presence of Ca^2+^. This shows *Al *binds* CaM,* even in the presence of Ca^2+^, and CaM undergoes a conformational change into an inactive form. Equilibrium dialysis and atomic adsorption studies indicated that Ca^2+^ remained bound to CaM simultaneously with *Al*. When the *Al*-chelator citrate was added to the solution only partial restoration of CaM activity occurred, suggesting that some of the *Al* ions became inaccessible for chelation [62]. 

The effects of* Cadmium (Cd^2+^)* were observed *in vivo,* where adult male rats, received 6 mg *Cd^2+^*/kg body weight daily for four weeks. Brain CaM activity was determined by measuring the stimulation of phosphodiesterase activity. A significant decrease in the CaM activity was observed after *Cd^2+^* treatment. CaM bound to *Cd^2+^* was also detected in the brains of rats exposed to *CdCl_2_*. It was proposed that, since *Cd^2+^* has an ionic radius similar to Ca^2+^, it might interact with the Ca^2+^-binding sites on the CaM [58, 61].

*Lead *was also found to interfere with CaM activity *in vitro *and* in vivo. In vitro* incubation of CaM with *lead (Pb^2+^) *increased the activity of calmodulin in terms of its ability to stimulate cAMP phosphodiesterase and a maximum increase was observed at 30 *μ*M lead concentration whereas at higher concentrations the calmodulin activity was inhibited. CaM-dependent cAMP phosphodiesterase activity increased up to a concentration of 100 *μ*M, following which there was a sharp decline in activity with higher concentrations of *lead*. The involvement of phenomenon of mimicry of calcium by *lead* as a mechanism of toxicity has been proposed. The affinity of *lead* to CaM is stronger than that of calcium and *lead* can displace calcium from calmodulin [59]. An *in vitro* study done on CaM purified from bovine brain showed that *Pb^2+^* mimics a natural ligand and raises the maximal activation slightly above the activation by Ca^2+^ [232].

#### 4.1.2. Protein Kinase C (PKC)

Protein Kinase C is a Ca^2+^ and phospholipid-dependent serine/threonine kinase that is a receptor for DAG and phorbol esters. There are two classes of PKC. The classical group of PKC consisting of four isozymes: PKC-*α*, PKC-*β*I, PKC-*β*II, and PKC-*γ*, are Ca^2+^-dependent and require Ca^2+^ as well as DAG or phorbol ester for their activation. The second group of PKC isoforms consists of five isozymes: PKC-*δ*, PKC-*ε*, PKC-*η*, PKC-*θ*, and PKC-*μ*. These do not require Ca^2+^ for their activation by DAG or phorbol ester. Various isozymes of PKC are involved in the formation of LTP. For instance, a null mutation in PKC-*γ* prevented the induction of LTP [233]. PKC is activated postsynaptically when metabotropic glutamate receptors (mGluR) are activated leading to the formation of DAG and release of intracellular Ca^2+^, which activates PKC. The mGluR- PKC pathway then increases the number and activity of AMPA receptors [224]. The PKC activity is affected by metal ions such as *Al^3+^, Pb^2+^, Hg,* and organic metals such as *methylmercury (MeHg; *refer to Figure 5).

*Aluminum (Al^3+^; *0–100 *μ*M) decreased* in vitro* PKC activity (determined by transfer of ^32^P from *γ*-^32^P-ATP to lysine rich histone in the presence of Ca^2+^ and phosphatidyl serine), and this effect was concentration dependent [20]. *In vivo*, rats fed *aluminum (AlSO_4_)* orally were found to have more PKC in the particulate fraction of the brain homogenate compared to the soluble fraction. Normally PKC is translocated from the cytosol to the membrane when it is activated. Application of *Al^3+^* caused a 70% increase in the total activity of PKC resulting in a greater fraction of it being translocated to the membrane, and hence the presence of greater fraction of PKC in particulate fraction compared to the soluble fraction [69].

*Lead acetate* upon *in vitro* incubation with PKC from adult rat brains significantly inhibited PKC activity with an IC_50_ of 2.12 *μ*M [64]. However, it was found that while very low concentrations of *Pb^2+^* (10^−13^ to 4 × 10^−4^ M) increased PKC activity, higher *Pb^2+^* concentrations (>4×10^−4^ M) caused an inhibition of PKC activity. When recombinant human PKC iso-enzymes were examined, low concentrations of *Pb^2+^* had very little activating effect on PKC-*γ* but inhibited it at higher concentrations (>4 × 10^−4^ M) [70]. *In vivo*, on exposure of adult rats to 1500 ppm *lead acetate*, there was a decrease in protein expression of PKC*γ* by 32% in the cytosol of hippocampal cells and 25% in the membrane fraction [68]. Another study comparing the effects of *Pb^2+^* on the PKC in the brain *in vivo* and *in vitro *found a considerable increase in PKC activity *in vitro, but* failed to find a considerable change in PKC activity *in vivo* [234]. 

*In vivo methylmercury chloride* administration in rats in five doses of 10 mg/kg body weight leads to a decrease in the enzymatic activity of the cytosolic PKC extracted from the brain, although it did not induce any change in second messenger binding as measured by binding of [^3^H]PDBu [67]. 

#### 4.1.3. Ca^2+^/Calmodulin Kinases

There are two types of Ca^2+^/calmodulin kinases (CaMK) involved in LTP: CaMKII and CaMKIV. The Ca^2+^-CaM complex generally activates these kinases. CaMKII is a serine/threonine protein kinase consisting of 12 subunits that are activated when activated calmodulin is associated with them [235]. Studies have shown that CaMKII blockers impede the ability to generate LTP. CaMKII can also be autophosphorylated at Thr^286^ and its activity becomes independent of Ca^2+^-CaM. This autophosphorylation occurs after the formation of LTP. It is suggested that after activation, CaMKII phosphorylates the AMPA receptor subunit as well as GluR1 and NMDA receptors and therefore enhances their conductance [236, 237].

CaMKIV is also activated similarly but the downstream targets are different for CaMKIV. Experiments have shown that upon activation, CaMKIV can phosphorylate CREB, which in turn mediates the transcriptional control of protein synthesis required for the long-term maintenance of LTP [238].

*In vivo*, rats exposed to 4 ppm *arsenic trioxide (As_2_O_3_)* for 60 days showed about a 4-fold decrease in expression of CaMKIV compared to a control group of rats as elucidated by microarray analyses. Western blot analyses reflected similar findings. Moreover, the decrease in expression of the *β*-subunit of CaMKIV was greater than the decrease in *α*-subunit expression [77]. 

#### 4.1.4. Nitric Oxide Synthase

Nitric oxide synthase (NOS) is an enzyme that produces nitric oxide (NO) by oxidizing L-arginine using molecular oxygen and NADPH [239, 240]. There are different kinds of NOS expressed in several cell types. Endothelial cells express constitutive endothelial NOS (eNOS) that is activated by Ca^2+^. Macrophages express inducible NOS (iNOS), and its expression is inducible by cytokines. Neurons express constitutive Ca^2+^-activated neuronal NOS (nNOS). Following the activation of NMDA receptors and the influx of Ca^2+^, it is believed that nNOS produces NO, a retrograde signal, that diffuses into the presynaptic membrane to enhance presynaptic neurotransmitter release by the production of cGMP during the formation of LTP [241, 242]. 

*In vivo*, chronic exposure to *aluminum* (*Al^3+^*) resulted in the reduced formation of NO after activation of NMDA in the rat cerebellum, as a consequence of decreased calmodulin and NOS [71].

*In vivo arsenic *exposure to 37 ppm sodium *arsenite *for 10 days, reduced NMDA-induced NOS activity (as measured by sampling of extracellular fluid by means of microdialysis). The maximal NMDA-induced increase of NOS activity (estimated by measuring the changes in extracellular citrulline in the exposed groups) was only 170 ± 24% while under control conditions it reached 278 ± 27% (*P*< 0.001) [72].

*In vitro* incubation of NOS with 100 *μ*M of *cadmium (CdCl_2_)* resulted in a significant reduction in brain NOS activity as measured by the conversion of radioactive arginine to citrulline. When incubated, the activity of NOS was decreased with an IC_50_ value of 0.22 mM [73]. 

*In vitro* incubation of NOS with *lead. *inhibited NOS activity with an IC_50_ of 0.36 mM [73]. *In vivo*, the cNOS activity in the hippocampus and cerebellum (measured by citrulline radioactivity following incubation with radioactive arginine) was decreased in rats that were exposed to 125, 250 and 500 ppm *lead acetate* for 14 days. This decrease was completely reversible by increasing the free Ca^2+^-concentration. The decrease in NOS activity correlated with blood lead levels [74].

#### 4.1.5. Extracellular Signal-Regulated Kinases (ERK1/2)

**Figure 6:**
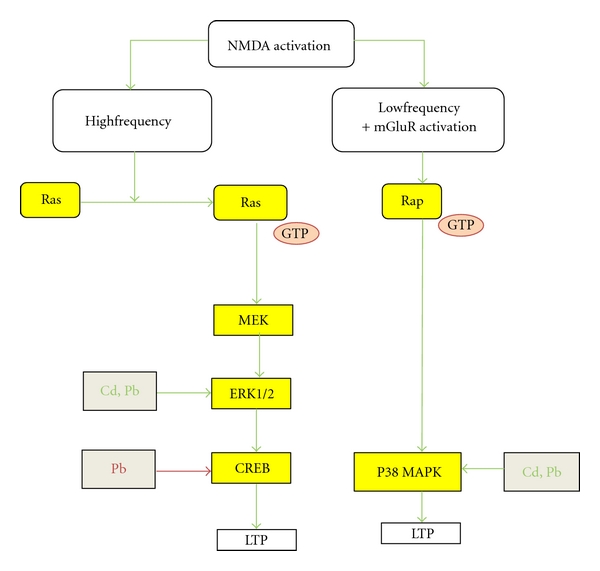
Molecules involved in the transcriptional control of LTP and LTD and effects of metals. LTP-3 depends on the activation of ERK1/2 and CaM kinase IV, which in turn phosphorylate CREB, and this leads to new protein synthesis. Other factors such as p38 mitogen-activated protein kinase (p38 MAPK) are involved in the formation of long-term depression (LTD) and c-JUN-N-terminal kinase (JNK) is thought to participate in LTD. A green color indicates an activation or an increase, and a red color indicates an inhibition or a decrease.

The extracellular signal-regulated kinases (ERK1/2) are serine threonine kinases that are activated when extracellular signals lead to an increase in intracellular Ras-GTP (GTP-bound form of Ras). Ras-GTP produced by an increase in guanyl nucleotide exchange factors (GEF), a decrease in activity of GTPase-activating proteins (GAPs) or a combination of both then leads to the activation of the enzyme MAPK/ERK kinase (MEK). MEK than activates ERK1 and ERK2 by phosphorylating them. ERK1 and ERK2 (also known as p44 and p42 MAPK) target transcription factors, cytoskeletal proteins, regulatory enzymes, as well as other kinases. In the postsynaptic membrane, calcium influx through the NMDA receptors leads to production of Ras-GTP that can then trigger the cascade leading to phosphorylation of ERK1 and ERK2. CREB maybe one of the targets of the ERK1/2 pathway involved in LTP [229, 243]. CREB, a member of the basic leucine zipper (bZip) family, is a transcription factor that is responsible for initiating new protein synthesis for the maintenance of L-LTP. PKA, CAMK and MAPK can activate CREB by phosphorylation at serine-133. On phosphorylation other proteins such as CREB binding protein are recruited to form a complex, which initiates transcription of CRE containing genes [244–250] (refer to Figure 6).

Exposure of hippocampal slices of rats to Cd^2+^ activates ERK1 and ERK 2 but only at very high concentrations (100–200 *μ*M CdCl_2_) [79, 229].

On incubation of ERK1/2 with 5 *μ*M Pb^2+^ *in vitro* for 3 hours, there was significant increase in ERK1 and ERK2 phosphorylation in hippocampal homogenates [80]. 

#### 4.1.6. P38 Mitogen-Activated Protein Kinase (p38 MAPK)

Parallel to the ERK1/2 pathway, which is involved in long-term potentiation, another MAPK cascade, which involves p38 MAPK is involved in long-term depression (LTD). Inhibition of p38 MAPK was shown to inhibit a form of hippocampal LTD that involved the activation of mGluR. Inhibition of ERK1/2 by blocking MEK had no effect on this form of synaptic plasticity. The pathways upstream and downstream of p38 MAPK are yet to be elucidated [229, 251] (refer to Figure 6).

Hippocampal slices of postnatal day 14 rats were exposed *to Cd^2+^* in concentrations between 5–100 *μ*M for 3 hours. A western blot analysis showed that this increased the activity of p38 MAPK, which is involved in the inhibition of LTP [79, 229].

Incubation of ERK1/2 with 5 *μ*M *Pb^2+^* for 3 hours resulted in a significant increase in p38 MAPK phosphorylation in hippocampal homogenates [80]. 

### 4.2. Disruption of Long-Term Potentiation by Exposure to Metals during Development

Developmental exposure to “neurotoxic” metals differs from exposure in an adult in various ways. The developing brain is more vulnerable than the adult one. The basic circuitry of the brain is laid down during development and any disruption of receptors, neurotransmission, and neurogenesis can prevent the brain from maturing normally. Inappropriate activation of the unspecific receptors in the developing brain can interfere with the normal “tuning.” Moreover, the blood-brain barrier is not laid down till approximately six months of age in humans. This absence of blood brain barrier allows toxic agents to enter the brain freely and interfere with its development. Developmental exposure to metals also raises the issue of what Costa et al. labeled as “silent” neurotoxicity. This is when the deleterious effects of various neurotoxic insults do not manifest until several months or years post-partum. For instance, in Guam's disease, unknown neurotoxic agents cause damage to the CNS, which do not become apparent until decades later. Here we discuss developmental exposure of metals and their effects on the molecules involved in the formation of LTP, which are important for the development of memory and learning [252]. 

N-methyl-D-aspartate receptors (NMDAR) are Ca^2+^ channels, which play an essential role in several forms of synaptic plasticity (see Section 3.1). They have glutamate receptors present which are involved in excitatory synaptic transmission in various parts of the brain. Its unique properties, such as Mg^2+^ block and high permeability to Ca^2+^, give NMDAR the ability to contribute to the formation of long-term potentiation and long-term depression. Several subunits of NMDA receptors have been identified: NR1 that is ubiquitously expressed; NR2 subunit family that has four distinct types (A, B, C, and D) and two NR3 subunits. The expression of the various subunits is different in different stages of development. For instance, NR2B and NR2D expression is present during the neonatal period and NR2A and NR2C are present in the later stages of development [253]. Due to its many binding sites (especially those for divalent cations), which change in their affinity to their agonists during development, a variety of (toxic non-physiologic) metals might bind to these NMDAR with a high affinity and thereby impair their function. 

*Lead* causes impairment of long-term potentiation in different regions of the hippocampus following chronic *lead* exposure [254, 255]. This has been associated with a disruption in the normal functioning of the NMDA receptors (NMDAR). NMDAR currents decrease after *in vitro* exposure to 5 *μ*M *lead* in hippocampal cells [152]. This can be attributed to the observation that *Pb^2+^* alters expression of the different subunits of NMDAR, which has been observed in the hippocampus and cerebral cortex. Additionally, a decrease in expression of NR2A subunit mRNA and proteins in the hippocampus have been seen [56, 57]. Also, the expression of NR1 subunit mRNA in the hippocampus and the cerebral cortex of rats increases [57], but this finding was not supported by another study by Nihei and Guilarte [56], which found no change in the expression of the NR1 subunit protein. 

*The effects of cadmium (Cd^2+^)* on calmodulin expression were determined in an *in vitro* study done on embryonic rat (ED 15) cerebral cortex, where the cortical slices were incubated with 10 nM *cadmium chloride* for 24 hours. This experiment showed a reduced the amount of calmodulin expression following *cadmium* exposure [63]. 

Nitric oxide synthase (NOS) was affected by the developmental exposure of rats to *aluminum (Al^3+^)* and *lead (Pb^2+^)*. Prenatal exposure of developing rats to *aluminum* sulfate (3%) decreased the content of neuronal NOS by 62 ± 12% in the cerebellum [76]. 

Perinatal exposure to *Pb^2+^* decreased NOS activity, as well as NOS expression. Chetty et al., using western blot analysis of nNOS in developing rat brain after perinatal exposure to 0.2% *lead* acetate, found a significant decrease in nNOS protein levels at postnatal day (PND) 21 and 35 in cerebellum, and at PND 21 in hippocampus [75].

Developmental *lead* exposure also affected PKC-*γ* and CaMKII function. PKC-*γ* is activated by binding of Ca^2+^ or DAG and on activation, it translocates to the membrane. To determine the effects of *Pb^2+^* on PKC-*γ* and other PKC-subtypes, pregnant rats were exposed to 0.1% *lead acetate*, dissolved in distilled deionized water (DDW) from gestation day 6 to postnatal day 21 (PND). With western blot analysis the expression on PKC-*γ* was determined. *Pb^2+^* reduced PKC-*γ* mRNA expression significantly in hippocampus and frontal cortex at PND 1, 5, and 10, with greater effect on the membrane PKC-*γ* than on the cytosolic PKC-*γ*. Additionally there was a decrease in the activity of PKC-*γ* following exposure to *lead*. The PKC-*γ* activity was determined by measuring the amount *γ*-^32^P transferred to histone per min per mg protein. In the hippocampus and the frontal cortex, both total and calcium-dependent PKC activities were significantly inhibited [65]. 

Moreover, rats exposed to 1500 ppm *Pb^2+^* during development demonstrated a reduction in the *V*_max⁡_ of CaMKII (examined by measuring the phosphorylation of a biotinylated substrate for CaMKII) and reduced expression of CaMKII *β* subunit in the hippocampus, but showed no changes in the sensitivity of calmodulin to CaMKII. In other words, the decrease in CaMKII activity was not due to impairment in its ability to bind CaM [78]. 

Various metals also inhibited the enzymes related to the transcription of new proteins involved in the formation of LTP. Two such targets are ERK1/2 and CREB.

Prenatal exposure to *aluminum* sulfate (3%) slightly increased the content of ERK [76]. Also, *in vivo* developmental exposure to 2 mg/kg of *Pb^2+^* increased both ERK1 and ERK2 phosphorylation in rat hippocampal neurons [80].

On developmental exposure to 1500 ppm of *Pb^2+^*, a decrease in the amount of phosphorylated CREB was observed in both the hippocampus (25% decrease) and the cerebral cortex (25% decrease) but there were no significant changes in unphosphorylated CREB levels [81]. Also, significant changes in the binding kinetics of CREB to CRE were observed in the hippocampus. The *K*_d_ and *B*_max⁡_ both were decreased by 38% and 30%, respectively, in the hippocampus but no significant changes in binding kinetics were observed in the cortex [244].

### 4.3. Summary of Long-Term Effects

Metals affect various mediators of synaptic plasticity. Calmodulin (CaM) activity is affected by *aluminum, cadmium,* and inorganic *lead*. Both *aluminum* and *cadmium* inhibited CaM activity [20, 58, 60–63] whereas inorganic *lead* first increased CaM activity at lower concentrations (possibly by mimicking calcium), but then at higher concentrations it decreased CaM activity [59, 232]. CaM is the central modulator of NMDAR-mediated synaptic plasticity and a majority of the regulators of synaptic plasticity depend on CaM for their activation. Thus, interference with CaM function will indirectly affect the function of numerous LTP- and LTD-related proteins such as adenylyl cyclase, Ca^2+^/Calmodulin kinases, nitric oxide synthase, and Ca^2+^ channels [226]. *Al^3+^*, *Pb^2+^,* and *MeHg *affect PKC activity, which is involved in the formation of LTP-1. *In vitro* studies demonstrated that in rat brain, *Pb^2+^* inhibited PKC activity at low concentrations but increased PKC activity at higher concentrations. However, when recombinant human PKC-*γ* was used, an opposite trend was observed [70]. Exposure to Pb^2+^ *in vivo *resulted in a decrease in protein expression whereas *MeHg* decreased the activity of PKC. *Al^3+^* inhibited PKC activity *in vitro* but ironically, oral administration lead to increase in PKC activity [20]. *Arsenic trioxide* (As_2_O_3_) decreases the expression of Ca^2+^/calmodulin kinase IV (CaMKIV) with a greater decrease in the *β*-subunit than the *α*-subunit *in vivo* [77]. Nitric oxide synthase (NOS) activity is decreased by various metals such as *Al^3+^, As^2+^, Cd^2+^,* and *Pb^2+^* [71–73]. The components of the transcription pathway, p38 MAPK and ERK1/2 were phosphorylated more when incubated with *Pb^2+^* and *Cd^2+^*. As discussed earlier, P38 MAPK is involved in the induction of LTD whereas ERK1/2 is involved in induction of LTP-3 [79, 80, 229].

## 5. Discussion and Conclusion

Most metals act on multiple modulators of synaptic transmission. Heavy metals such as *mercury, lead,* and *arsenic* interfere with normal functioning of molecules both presynaptically and postsynaptically. They also target molecules involved in synaptic plasticity. As discussed above *in vivo* and *in vitro* studies have shown that metals inappropriately inhibit or activate various molecules involved in synaptic transmission and synaptic plasticity. Even though the current pool of the literature gives us valuable insights into the mechanisms of metal toxicity at the synapse, there are many limitations of the current studies.

Firstly, there is hardly any sufficient information with regard to the effect of metals at ***different stages of development***. Postsynaptically, there is a strong suggestion that the different effects on development are due to different subunit expression. As discussed in Section 3.2, *Pb^2+^* was a more potent inhibitor of Glu-activated currents in NMDAR expressing NR2A or NR2B compared to receptors expressing both these subunits [157]. At the same time, *Pb^2+^* showed high- and low-affinity components for its inhibition in PN14 and PN21 hippocampal membranes. These data suggested that the high-affinity *Pb^2+^*-sensitive site was associated with receptors expressing NR2A or NR2B subunits, while the low-affinity site was associated with receptors expressing both subunits (to see the effects on other brain areas, review [153]). The support to this hypothesis came from studies showing that the developmental pattern of NR2A and NR2B mRNA in the hippocampus was similar to that in the data presented [256].

This developmental aspect is not well studied for most of the metals and also for AMPA/kainate and GABA receptors. The effects of divalent metals on AMPA/kainate receptors seem to be dependent upon the subunit composition as well, particularly the presence of GluR2 subunit which rendered the channel impermeable to Ca^2+^. *Zn^2+^* and *Co^2+^* both had dual effects on AMPA-Rs: at micromolar concentrations they enhanced AMPA receptor responses whereas at millimolar concentrations, they had inhibitory actions. Various metals caused inhibition of Ca^2+^ impermeable AMPA-R, the inhibition was fast, reversible, and voltage independent. The rank order of activities was *Ni^2+^ > Zn^2+^*>* Co^2+^*>* Ca^2+^*>* Mn^2+^*>* Mg^2+^* [190]. The proposed mechanism of action was that complexes of AMPAR agonists and divalent cations compete with the free agonists for the binding sites.

***Prenatal exposure*** to heavy metals also leads to various changes in the LTP machinery in the developing brain as discussed before. *Lead*, for instance, changed the expression pattern for NMDAR subunits, and decreased the expression and activity of PKC-*γ*, CaMKII *β*, and nNOS in various areas of the brain. These results could explain why the development exposure to some metals causes cognitive deficits in children.

Moreover, a majority of the studies were done *in vitro*, and the *in vivo* studies were done in rats. In most *in vitro *studies, either brain homogenates or purified target molecules were incubated with a given metal. These studies therefore may not accurately depict the physiologic effects of the metals since they do not undergo the physiological process of absorption from the gut as in the human body, and the alterations that may occur in the blood before the metals reach the target tissue. Also, the solutions used for the preservation of the cells may interfere with the experimental results rendering them inaccurate. Moreover, the contents of the media and the forms of metals used were inconsistent between studies. Consequently properties such as solubility of metals in the media and presence of anions and pH were variable and beyond the scope of this study to discuss. It is definitely a limitation of *in vitro* studies, which makes it difficult to compare the different experiments, even when an identical concentration of the same metals was used. However, the ease of carrying out the experiment and lack of requirement for storage space for animals make them a likely choice for most researchers. *In vivo* studies may be closer to the physiologic processes; however it becomes harder than to vary the concentrations of metals and monitor them at the selected site of interest.

In addition, most of the findings presented in this review both *in vivo* and *in vitro* were based on studies done using rats. Even though it is easy to measure concentrations of metals, levels of proteins and enzymes in rats, elucidating the clinical manifestations in animal models can be challenging. Also, it is hard to find whether the effects in rats are similar to those in humans and if the effects in rats are representative of the effects in humans. Higher cognitive functions in humans might alter the presentation of the toxicity in ways that cannot be adjusted for because much of the mechanisms of the functioning of the human nervous system are not fully understood today.

Another important limitation of the currently available literature is that the majority of studies discussed were on effects of inorganic metals on the brain cells or brain molecules, and very few centered around organic metals which are perhaps even more significant than inorganic metal toxicity since in some cases the organic forms are more toxic than the inorganic forms, as for *mercury* [257]. There is a rapidly growing body of evidence that the majority of metals may actually be methylated to their organic form as the body attempts to detoxify metals. For *arsenic*, in the past it was believed that conversion of arsenic to *monomethyl arsenic *and* dimethyl-arsenic* was a method of detoxification; however, the view has changed since then with the recognition that methylated metabolites of trivalent arsenic are carcinogenic [258].* Antimony, mercury, lead, tin, and selenium* are known to cause public health problems in their methylated forms. *Cadmium, cobalt, mercury, and nickel* reportedly undergo biomethylation; however, the effects of biomethylation have been studied more in unicellular organisms rather than plants and animals, therefore it has been suggested that although biomethylation does occur in plants and animals, the rates are likely to vary on the basis of the animal and the metal, and its concentration [259].

There are some situations that have not been considered in most experimental designs. One such issue that arises with the study of metals is the problem that most studies are not reflective with regard to the actual exposure in nature where humans are simultaneously exposed to more than one metal. Very few studies have targeted this issue, most likely due to the complicated nature of conducting an experiment with many variables and determining the contribution of each. One study by Platt and Büsselberg who examined the effects of combinations of *Pb^2+^*, *Zn^2+^,* and *Al^3+^*, on voltage-activated calcium channels by simultaneous application of various combinations of two metals determined that regardless of the order in which the metals were added, the actions were in fact additive [260]. Whether this is the case with other metals is not certain and there is not enough data in the literature describing effects of combinations of metals. Another limitation of the study is that some targets may not be as relevant as others in causing the clinical symptoms of metal toxicity; however, it is not possible to know at this stage the exact contribution of each target. 

Finally, the most important objective is to put these effects of metals into practical use. This can be done by using the data of the toxic concentrations of metals to make a meaningful decision in regard to their acceptable blood levels. There is evidence to suggest that currently accepted levels for some metals are still not “safe” levels. Even at the currently accepted blood *lead* levels of 10 *μ*g/dL, it is causative of preterm labor and adverse pregnancy outcome [261]. Therefore, there is a need to reevaluate the accepted blood concentrations of metals in light of the newer evidence as it appears. 

Metal neurotoxicity is a field, which is abounding with the literature and excellent research; however, in the current literature some metals are highlighted while for other metals (or metal compounds) hardly any data are available. There is an emphasis on certain metals such as *lead*, whose harmful effects are well known while there is very little known about certain metal groups such as *lanthanides* and *actinides*. 

There are certain targets where metal actions have been excessively examined such as voltage-activated calcium channels while there is little known about the effects of metals on parts of signaling pathways such as phosphodiesterases and IP_3_. This raises the need to evaluate new targets for metals, which have not been studied before, which may prove to have a groundbreaking effect in the field of neurotoxicity.

To summarize, exposure to different metals occurs due to industrial activities, environmental, and food chain contamination. This paper elucidated the various targets of metals in synaptic transmission and synaptic plasticity. Exposure to metals had varied effects on different synaptic targets, which were dependent on the form of metal, the concentration of metal, route of exposure (*in vitro* or *in vivo*), the medium used, and even the duration of exposure in some cases. 

## Figures and Tables

**Table 1:** Effects of metals on voltage-activated ion channels (↑—activation/upregulation,  ↓—inhibition/downregulation).

	Voltage-gated channels											
Target		Calcium channels						Sodium channels			Potassium channels	
		L	N	T	R	All^(ii)^		Tetrodotoxin sensitive	Tetrodotoxin resistant	All^(i)^		
Al	Effect	↓	↓			↓						
	Conc	20 *μ*M	20 *μ*M			50 *μ*M						
	Ref	[1]	[1]			[2]						

Cd	Effect		↓			↓		↓	↓			
	Conc		20 *μ*M			2.2, 125 *μ*M		5 mM	0.2 mM			
	Ref		[3]			[4], [5]		[6]	[6]			

Co	Effect					↓						
	Conc					500 *μ*M						
	Ref					[7]						

Hg	Effect		↓	↓	↓	↓						
	Conc		2.2 *μ*M	0.5–2 *μ*M	0.7 *μ*M	1.1 *μ*M						
	Ref		[8]	[9]	[8]	[10]						

La	Effect										↑	
	Conc										10 *μ*M	
	Ref										[11]	

Mn	Effect										↓	↑
	Conc										10 mM	1 mM
	Ref										[12]	[12]

Pb	Effect	↓	↓	↓		↓						
	Conc	30 nM^(i)^, 0.7, 0.64, 0.1 *μ*M	80 nM^(i)^, 0.64, 0.1 *μ*M	1.3 *μ*M, 6 *μ*M, 6 *μ*M		1, 1, 0.6, (1–30) *μ*M						
	Ref	[13], [14], [15], [1]	[13, 15] [1], [5], [1]	[14], [15], [4]		[16], [15], [4], [17]						

Pt	Effect					↓				↓	↓	
	Conc					23.9 *μ*M				10 *μ*M	10 *μ*M, 100 *μ*M	
	Ref					[18]				[18]	[18], [18]	

Sn	Effect					↓	↑					
	Conc					50 *μ*M	50 *μ*M					
	Ref					[19]	[20]					

Zn	Effect	↓	↓	↓		↓		↓	↓		↑	
	Conc	5,69 *μ*M	5,69 *μ*M	20 *μ*M		2 mM, 69 *μ*M		2 mM	50 *μ*M		30 *μ*M	
	Ref	[1, 21]	[1, 21]	[21]		[10, 16]		[6]	[6]		[22]	

^(i)^Extent of block and EC_50_ differ based on concentration of charge carrier used.

^(ii)^Paper does not describe which sub-type is affected.

**Table 2:** Effects of metals on presynaptic signaling pathways (↑—activation/upregulation, ↓—inhibition/downregulation).

Target		Pathways							
		PKC		Adenylate cyclase		Phosphodiesterase	CaM	IP3	Intracellular calcium
Al	Effect	↓	↑				↓		↑
	Conc	0–100 *μ*M					0–1000 microM		0–1000 *μ*M
	Ref	[2]	[23]				[2]		[2]

As	Effect								↑
	Conc								1 *μ*M
	Ref								[24]

Cd	Effect				↓	↓			↑
	Conc				0.4, 0.9, 1.4^ (i)^ *μ*M				0.1–1 mM
	Ref				[25], [25], [25]	[25]			[26]

Hg	Effect				↓	↓			
	Conc				0.8, 0.5, 0.9^ (i)^ *μ*M				
	Ref				[25], [25], [25]	[25]			

Ni	Effect	↓							
	Conc	30 *μ*M							
	Ref	[27]							

Pb	Effect			NC	↓	↓			
	Conc			1500–10000 ppm	2.5, 8.6,1.9, 8.0^ (i)^ *μ*M				
	Ref			[25]	[28], [25], [25], [25]	[25]			

Pt	Effect							↑	↑
	Conc							1 nM–10 *μ*M	1 nM–10 *μ*M
	Ref							[29]	[29]

Zn	Effect				↓				
	Conc				1-2 *μ*M				
	Ref				[30]				

^(i)^The three different concentrations indicate actions in different areas of the brain—cerebrum, cerebellum, and brainstem.

**Table 3:** Effects of metals on transporters, neurotransmitters, and neuropeptides (↑—activation/upregulation, ↓—inhibition/downregulation).

Target		Transporters				Neurotransmitters						Neuropeptides
		Ca^2+^ into mitochondria		Ca^2+^ ATPase	Dopamine transporter	Glutamate	Aspartate	GABA	Glycine	Dopamine	Acetylcholine	Substance P, neuropeptide K, and neurokinin
Al	Effect			↓								
	Conc			0–100 *μ*M								
	Ref			[2]								

Cd	Effect					↓^( v )^	↓^( v )^	↑^(v)^	↑^(v)^			↓
	Conc					10–30 *μ*M	10–30 *μ*M	10–30 *μ*M	10–30 *μ*M			5^(iii)^ *μ*M
	Ref					[31]	[31]	[31]	[31]			[32]

Cu	Effect									↓		
	Conc									10^(i)^ *μ*g m^−1^		
	Ref									[33]		

Hg	Effect				NC					h		
	Conc				400 *μ*M					6 mg/kg, 400 *μ*M		
	Ref				[34]					[35], [34]		

Mn	Effect					↓	↓	↓		↓		
	Conc					20–200 nm^(iv)^	20–200 nm	20–200 nm		10^(ii)^ *μ*g mL^−1^		
	Ref					[36]	[36]	[36]		[33]		

Ni	Effect									↑		
	Conc											
	Ref									[37]		

Pb	Effect					↑		↑			↑	
	Conc							50 *μ*mol L^−1^			100 *μ*mol L^−1^	
	Ref					[38]		[39], [38]			[39]	

Pt	Effect	↓	NC									
	Conc	0.5 mM	500 *μ*M									
	Ref	[40]	[41]									

Sn	Effect										↑	
	Conc										10–100 *μ*M	
	Ref										[42]	

^(i)^Upregulates expression of monoamine oxidase, decreases production, and increases depletion

^(ii)^Downregulates expression of tyrosine hydroxylase gene

^(iii)^Reduces expression of precursor gene

^(iv)^Another study shows that uptake of glutamate into astrocytes is reduced through the decreased expression of glutamate aspartate transporter; this may result in increase in glutamate levels in the synapse [43]

^(v)^Shows effect on neurotransmitter release.

**Table 4:** Effects of organic metals on presynaptic targets (↑—activation/upregulation, ↓—inhibition/downregulation).

Target			MeHg			Me_3_Pb			Et_3_Pb			Me_3_Sn			Et_3_Sn		
			Effect	Conc	Ref	Effect	Conc	Ref	Effect	Conc	Ref	Effect	Conc	Ref	Effect	Conc	Ref
Voltage-gated channels	Calcium channels	L															
		N	↓	1.3 *μ*M	[8]												
		T															
		R	↓	1.1 *μ*M	[8]												
	All^(i)^		↓	0.25–1 *μ*M	[44]	↓	0.5–50 *μ*M	[45]									
	Potassium channels		↓	2.2 *μ*M	[46]												

ATPases															↓	260 *μ*M	[47]
	Na^+^K^+^ATPase								↓	5–20 *μ*M	[48]	↓	5–20 *μ*M	[48]	↓		

Transporters	Na^+^-dependent GABA transporter								↓	10 *μ*M	[49]	↓	10 *μ*M	[49]	↓		

Pathways	IP_3_		↑	3 *μ*M	[50]												
	Intracellular Calcium		↑	(0.5–1) *μ*M	[51]												
	Synapsin I											↓		[52]			
	p38											↓		[52]			

Neurotransmitters	GABA								↑^(iii)^	10 *μ*M	[49]	↑^(ii)^	75 *μ*M	[53]	↓		
	Dopamine											↓		[54]			
	Norepinephrine											↑^(ii)^	43 *μ*M	[53]	↓		
	Serotonin											↑^(ii)^	24 *μ*M	[53]	↓		

^(i)^Paper does not describe which subtype is affected

^(ii)^Decreases uptake of neurotransmitter into synaptosomes, thereby probably increasing the amount in cleft

^(iii)^Release of neurotransmitter from vesicles is being measured.

**Table 5:** Effects of metals on postsynaptic ligand-gated ion channels (↑—activation/upregulation, ↓—inhibition/downregulation).

Target		NMDA	AMPA/kainate	GABA-A
Lead	Effect	(1) ↓ open channel probability		(1) ↓ (35%)
		(2) 60% ↓ in current (reversible)		
		(3) >80% block		
		(4) ↓		
		(5) ↓		
		(6) ↓ receptor binding		
		(7) ↓		
			
	Conc	(1) 1–10		(1) 1m M
		(2) 50		
		(3) 100		
		(4) IC_50_ = 1.52–8.19		
		(5) IC_50_ = 8.78 (in 0 Zn); IC_50_ = 1.26 (10 Zn) at high site, 94 at low site		
		(6) IC_50_ = 300 (adult); 60 (neonatal)		
		(7) IC_50_ (free) = 0.55		
			
	Ref	(1) [16]		(1) [10]
		(2-3) [14]		
		(4) [20]		
		(5) [9]		
		(6) [12]		
		(7) [5]		

Zinc	Effect	(1) ↓ open	(1) ↑	(1) ↓
		channel probability		
		(2) Channel block	(2) ↓	↓ current in voltage independent, noncompetitive manner
		(3) NR2A block	(3) ↑	
		(4) NR2B block	(4) ↓	
		(5) ↓	(5) ↑ (16% to kainate, 15% to glu peak and steady state)	
		(6) ↓ receptor binding (76%)	(6) ↓	
		(7) ↓	(7) ↑ AMPA response	
		(8) ↓	(8) ↑ desensitized Kainate responses	
			(9) ↓ AMPA and kainate responses	
			
	Conc	(1) 1–10 *μ*M	(1) 50	(1) 100 (dose dependent)
		(2) >20	(2) 1 mM	(2) IC_50_ = 19
		(3) nM	(3) <300	
		(4) *μ*M	(4) >500	
		(5) High affinity: IC_50_ = 0.77; low affinity: IC_50_ = 153	(5) 10	
		(6) 1 mM	(6) IC_50_ = 700	
		(7) IC_50_ (free) = 1.3	(7) EC_50_ = 30	
		(8) IC_50_ = 42.9	(8) EC_50_ = 13	
			(9) IC_50_ = 1.2-1.3 mM	
		
		(1-2) [11]	(1-2) [17]	(1) [13]
	Ref	(3) [17, 21]	(3-4) [7, 25]	(2) [10]
		(4) [17]	(5-6) [15]	
		(5) [9]	(7-9) [22]	
		(6) [12]		
		(7) [5]		
		(8) [22]		

Magnesium	Effect	(1) ↑ NMDA-R affinity to glycine in all receptors	(1) ↓ (27%)	
		(2) ↓ elementary current at +ve potentials(+20 to +80)		
		(3) ↑ glycine and voltage-independent and subunit specific		
		(4) external channel block, voltage dependent		
			
	Conc	(1) 10 mM	(1) 20 mM	
		(2) 10 mM		
		(3) 2 mM		
		(4) IC_50_ (−100 mV) = 2–15		
			
	Ref	(1–3) [6]	(1) [15]	
		(4) [2]		

Manganese	Effect	(1) ↓ (activity dependent, channel blocker)	(1) ↓ (46%)	(1) Little or no effect
			
	Conc	(1) Ki = 35.9 (presence of glu and gly); Ki = 157 (no glu nor gly)	(1) 25 mM	(1) 1 mM
			
	Ref	(1) [8]	(1) [15]	(1) [10]

Copper	Effect	(1) ↓	(1) ↓	(1) ↓ (voltage independent)
		(2) ↓ receptor binding (54%)	(2) ↓ kainite-induced current	
		(3) ↓ (channel block)	(3) ↓ efficacy of kainate	
		(4) ↓		
		(5) ↓ voltage independent, noncompetitive		
			
	Conc	(1) ND	(1)	
		(2) 1 mM	(2) IC50 = 4.3	
		(3) Ki = 195 (no coagonists);	(3) 30	
		two sites (9.4, 248) with glu and gly		(1) IC_50_ = 5
		(4) IC_50_ = 15		
		(5) IC_50_ (free) = 0.27		
			
	Ref	(1) [17, 23]	(1) [17]	(1) [10]
		(2) [12]	(2-3) [30]	
		(3) [8]		
		(4) [30]		
		(5) [28]		

Cobalt	Effect	(1) ↓	(1) ↓	(1) ↓
			(2) ↓	(2) ↓ (29%)
			
	Conc	(1) 2 mM	(1) 2 mM	(1) 2 mM
			(2) IC_50_ = 6.1 mM	(2) 1 mM
			
	Ref	(1) [3]	(1) [3]	(1) [3]
			(2) [15]	(2) [10]

Nickel	Effect	(1) NR2A: ↓, NR2B: ↑	(1) ↓ (kainite-induced current)	(1) ↓(20%)
		(2) NR2A ↓ (100% at +ve potentials)	(2) ↓ (glu-induced current)	
		(3) NR2B ↓		
		(4) NR2B ↑ (voltage independent)		
			
	Conc	(1) 30		
		(2) IC_50_ = 36 at −60 mV and 81 at +40 mV		
		(3) IC_50_ 138 at −60 mV and 442 at +40 mV		
		(4) 3		
			(1) IC_50_ = 420	(1) 1 mM
			(2) IC_50_ = 2.6 mM	
			
	Ref	(1) [16]	(1-2) [15]	(1) [10]
		(2–4) [21]		

Mercuric chloride	Effect			(1) ↑ 130%
				(2) ↑ (270%)
			
	Conc			(1) 1
				(2) 100
			
	Ref			(1) [1]
				(2) [19]

Methyl mercury	Effect	(1) ↓ receptor binding		(1) ↓ amplitude to 82.4%
			
	Conc	(1) IC_50_ = 0.95 (neonatal); 70 (adult)		(1) 100
			
	Ref	(1) [12]		(1) [55]

Cadmium	Effect	(1) ↓ receptor binding (58%)	(1) ↑ (kainate to 108% and QA to 115%)	(1) ↓ (18%)
		(2) ↓ (39% of control)	(2) ↓ (kainate to 79% and QA to 60%)	
		(3) ↓ (4% of control)		
			
	Conc	(1) 1 mM	(1) 50	(1) 1 mM
		(2) 50	(2) 1 mM	
		(3) 1 mM		
		(1) [12]	(1-2) [18]	(1) [10]
	Ref	(2-3) [18]		

Lanthanide	Effect	(1) ↓ NMDA response in a voltage-independent manner	(1) ↑	(1) ↑ (300% max) and ↑ as the potential more −ve
			(2) ↓	
			
	Conc	(1) IC_50_ = 2	(1) 1–100	(1) EC_50_ = 231
			(2) >100	
			
	Ref	(1) [27]	(1-2) [27]	(1) [10]

Trimethyl-tin (TMT)	Effect	(1) ↓ (35%) reversible	(1) ↓ (20%) irreversible	(1) ↓ (30%) irreversible
			
	Conc	(1) 100	(1) 100	(1) 100
			
	Ref	(1) [4]	(1) [4]	(1) [4]

**Table 6:** 

																
Target		Al			As			Cd			Hg			Pb		
		Effect	Concentration	Ref	Effect	Concentration	Ref	Effect	Concentration	Ref	Effect	Concentration	Ref	Effect	Concentration	Ref
Pumps	Ca^2+^ ATPase	↓*in vitro *	10 mg/kg/day Al^3+^	[1]												

Protein Synthesis	NMDAR NR1													*↕* dev.	750 ppm PbAc	[13]
														↑ dev.	750 ppm PbAc	[14]
	NMDAR NR2A													↓ dev.	750 ppm PbAc	[13]
														↓ dev.	750 ppm PbAc	[14]
	NMDAR NR2B													*↕* dev.	750ppm PbAc	[13]
														*↕* dev.	750 ppm PbAc	[14]

Enzymes	CaM	↓*in vitro *	10 mg/kg/day Al^3+^	[1]				↓*in vivo *	6 mg/kg/day CdCl_2_	[15]				↑ *in vitro *	30 *μ*M Pb^2+^	[21]
		↓*in vitro *	IC_50_ —15 *μ*M AlCl_3_	[3]				↓*in vitro *	IC_50_ —0.47 mM CdCl_2_	[8]						
		↓*in vitro *	AlCl_3 _—240 *μ*M	[9]				↓*in vitro *	10 nM CdCl_2_	[4]						
	PKC	↓*in vitro *	10 mg/kg/day Al^3+^	[1]							↓*in vitro *	IC_50 _—1.5 *μ*M Hg; IC_50_ — 0.2 2*μ*M CH_3_Hg	[2]	↓ dev.	0.1% PbAc	[5]
		↓*in vivo *	0.3% AlSO_4_ for 4 months	[18]							↓*in vitro *	IC_50 _—0.08 *μ*M HgCl_2_; IC_50 _—1.32 *μ*M CH_3_Hg; IC_50 _—0.90 *μ*M C_6_H_5_Hg^+^	[7]	↓*in vitro *	IC_50_ —2.12 *μ*M PbAc	[10]
											↓*in vivo *	10 mg MeHg/kg	[16]	↓*in vivo *	1500 ppm PbAc	[17]
														↑ *in vitro *	10^−13^ to 4 × 10^−4^ M PbAc	[19]
														↓*in vitro *	> 4 × 10^−4^ M PbAc	
	NOS	↓*in vivo *	2.5% AlSO_4_ for 3–5 weeks	[20]	↓*in vivo *	37 ppm NaAsO_2_	[6]	↓*in vitro *	100 *μ*M CdCl_2_	[11]				↓*in vivo *	125 ppm PbAc	[12]
		↓dev.	3% AlSO_4_	[27]										↓ dev.	0.2 % PbAc	[22]
														↓*in vitro *	IC_50_—0.36 Pb^2+^	[11]
	CaMK II													↓dev.	1500 ppm PbAc	[25]
	CaMK IV				↓*in vivo *	4 ppm As_2_O_3_ for 60 days	[23]									

Transcription factors	P38 MAPK							↑ *in vitro *	5–100 *μ*M CdCl_2_	[28]				↑ *in vitro *	5 *μ*M Pb^2+^	[30]
	ERK1/2 pho-sphorylation							↑ *in vitro *	100–200 *μ*M CdCl_2_	[28]				↑dev.	2 mg/kg/day PbAc	[30]
														↑ *in vitro *	5 *μ*M Pb^2+^	[30]
	CREB phospho-rylation													↓ dev.	1500 ppm PbAc	[29]

Julka and Gill [20], Nihei and Guilarte [56], Guilarte and McGlothan [57], Vig et al. [58], Sandhir and Gill. [59], Siegel and Haug [60], Cox and Harrison [61], Levi et al. [62], Ohtani-Kaneko et al. [63], Rajanna et al. [64], Xu et al. [65], Inoue et al. [66], Rajanna et al. [64], Saijoh et al. [67], Nihei et al. [68], Johnson et al. [69], Coppi et al. [70], Hermenegildo et al. [71], Zarazua et al. [72], Mittal et al. [73], García-Arenas et al. [74], Chetty et al. [75], Llansola et al. [76], Wang et al. [77], Toscano et al. [78], Rigon et al. [79], Cordova et al. [80], Toscano et al. [81].

## Abbreviations

[Ca^2+^]_i_: Intracellular calcium concentration
ABD: Agonist-binding domain
AC: Adenylate cyclase
Ach: Acetylcholine
Al: Aluminum
AlCl_3_: Aluminum trichloride
AMPA: *α*-amino-3-hydroxy-5-methyl-4-isoxazolepropionic acid
As: Arsenic
As_2_O_3_: Arsenic trioxide
ATD: Amino terminal domain
ATP: Adenosine triphosphate
ATPase: Adenosine triphosphatase
Ba: Barium
BAPTA: 1,2-bis(2-aminophenoxy)ethane-N,N,N9,N9-tetracetic acid tetrakis(acetoxymethyl) ester
*B*_max⁡_: Maximal binding
Ca: Calcium
CaM: Calmodulin
CaMK: Ca^2+^/calmodulin-dependent protein kinase
cAMP: Cyclic adenosine monophosphate
Cd: Cadmium
CdCl_2_: Cadmium chloride
CDDP: *cis-Diammine-dichloroplatin*
CH_3_Hg: Methylmercury
CNS: Central nervous system
Co: Cobalt
Cr: Chromium
CRE: cAMP response elements
CREB: Ca^+2^/cAMP response element-binding protein
Cu: Copper
DAG: Diacylglycerol
DDW: Distilled deionized water
DOC2: Double C2 domain
DOPAC: 3.4-dihydroxyphenylacetic acid
DOPAC: 3.4-dihydroxyphenylacetic acid
DRG: Dorsal root ganglion
E-LTP: Early-phase LTP
ED: Embryonic day
eNOS: Eendothelial NOS
EPP: End-plate potential
ERK1/2: Extracellular signal-related kinase 1/2
Et_3_Pb: Triethyl lead
Et_3_Sn: Triethyl-tin
Fe: Iron
GABA: *γ*-Aminobutyric acid
GAP: GTPase-activating protein
GEF: Guanyl nucleotide exchange factors
G_i/o_: Inhibitory Sguanine nucleotide binding protein/other guanine nucleotide-binding protein
Glu: Glutamate
GluR: Glutamate Receptor
Gly: Glycine
GPCR: G-protein-coupled Receptors
Gpp(NH)p: 5′ Guanylylimidodiphosphate
G_s_: Stimulatory guanine nucleotide binding protein
GTP: Guanosine-5′-triphosphate
Hg: Mercury
HVA: Homovanillic acid
IC_50_: Concentration for 50% inhibition
iNOS: Inducible NOS
IP_3_: Inositol triphosphate
Inositol triphosphate: Inhibitory postsynaptic currents
IQ: Intelligence quotient
JNK: c-JUN-N-terminal kinase
K: Potassium
KCl: Potassium chloride
*K*_d_: Dissociation constant
L-LTP: Late-phase LTP
La: Lanthanum
Li: Lithium
LTD: Long-term depression
LTP: Long-term potentiation
mAb: Monoclonal antibody
MAPK: Mitogen-activated protein kinase
Me_3_Pb: Trimethyl lead
Me_3_Sn: Trimethyl-tin
MeHg: Methylmercury
MEK: MAPK/ERK kinase
MEPP: Miniature end-plate potential
Met: Methionine
Mg: Magnesium
mGluR: Metabotropic glutamate receptors
mIPSC: Miniature inhibitory postsynaptic currents
Mn: Manganese
mRNA: Messenger ribo nucleic acid
Na: Sodium
NADPH: Reduced nicotinamide adenine dinucleotide phosphate
Ni: Nickel
NMDA: N-methyl-D-aspartate
NMDAR: N-methyl-D-aspartate receptors
nNOS: Neuronal NOS
NOS: Nitric oxide synthase
NT: Neurotransmitter
Pb: Lead
PKA: Protein kinase A
PLC: Phospholipase C
PND: Postnatal day
Ras: Rat sarcoma family
RBC: Red blood cells
Sn: Tin
SNAP-25: Synaptosome-associated protein 25 kDa
SNARE: SNAP and NSF attachment receptor
SnCl_2_: Stannous chloride
Sr: Strontium
STP: Short-term potentiation
Thr: Threonine
VGCC: Voltage-gated calcium Channel
*V*_m_: Membrane voltage
Zn: Zinc.
